# A microbiota–root–shoot circuit favours *Arabidopsis* growth over defence under suboptimal light

**DOI:** 10.1038/s41477-021-00956-4

**Published:** 2021-07-05

**Authors:** Shiji Hou, Thorsten Thiergart, Nathan Vannier, Fantin Mesny, Jörg Ziegler, Brigitte Pickel, Stéphane Hacquard

**Affiliations:** 1 Max Planck Institute for Plant Breeding Research, Cologne, Germany; 2 Department of Molecular Signal Processing, Leibniz Institute of Plant Biochemistry, Halle, Germany; 3 Cluster of Excellence on Plant Sciences (CEPLAS), Max Planck Institute for Plant Breeding Research, Cologne, Germany

**Keywords:** Microbiology, Plant symbiosis

## Abstract

Bidirectional root–shoot signalling is probably key in orchestrating stress responses and ensuring plant survival. Here, we show that *Arabidopsis thaliana* responses to microbial root commensals and light are interconnected along a microbiota–root–shoot axis. Microbiota and light manipulation experiments in a gnotobiotic plant system reveal that low photosynthetically active radiation perceived by leaves induces long-distance modulation of root bacterial communities but not fungal or oomycete communities. Reciprocally, microbial commensals alleviate plant growth deficiency under low photosynthetically active radiation. This growth rescue was associated with reduced microbiota-induced aboveground defence responses and altered resistance to foliar pathogens compared with the control light condition. Inspection of a set of *A. thaliana* mutants reveals that this microbiota- and light-dependent growth–defence trade-off is directly explained by belowground bacterial community composition and requires the host transcriptional regulator MYC2. Our work indicates that aboveground stress responses in plants can be modulated by signals from microbial root commensals.

## Abstract (web summary)

A synthetic root microbial community rescues weak growth under low light and enhances immunity in *Arabidopsis*. Transcription factor MYC2 regulates both this coordination between rhizosphere and shoots and the growth/defence trade-off under low light conditions.

## Main

Unlike animals, plants are sessile organisms that must simultaneously integrate various responses to biotic and abiotic stresses to prioritize either growth or defence depending on rapidly changing surrounding conditions^1^. Since the colonization of land by ancestral plant lineages 450 million years ago, complex multikingdom microbial consortia have interacted with and colonized the roots of healthy plants^2^. Direct integration of microbial and environmental cues by plants is therefore probably key in orchestrating their ability to overcome environmental stresses^3–5^. The beneficial activities conferred by microbial root commensals have been primarily described in the context of nutritional stress^4,6–9^ or pathogen attack^10–14^, revealing that root colonization by complex microbial communities can shape host phenotypic plasticity and protect plants from diseases. By analogy with the microbiota–gut–brain axis described in animals, the concept of the microbiota–root–shoot axis was recently introduced in plants^15,16^, suggesting that bidirectional root–shoot signalling might represent a key mechanism linking root microbiota assembly to aboveground stress responses.

Carbohydrate biosynthesis, energy production and therefore plant growth depend on the amount and quality of photosynthetically active radiation (PAR) absorbed by chloroplasts in leaves^17^. Spectral PAR irradiance ranges from 400 to 700 nm under natural conditions, and a reduction of both the amount of PAR (light quantity) and the ratio of red to far-red light (light quality) is perceived as a warning signal in plants, thereby triggering adaptive changes in shoot morphology^18–21^. Light-dependent resource allocation has been shown to be tightly linked with defence signalling in *Arabidopsis thaliana* and tomato leaves, suggesting that aboveground responses to light and pathogens are interconnected^22–26^. However, the extent to which belowground commensals can modulate or even dictate aboveground stress responses remains unknown. Given the fact that a substantial proportion of photosynthetically fixed carbohydrates (that is, >15%) is invested in the rhizosphere^27,28^, we hypothesized that the aboveground response to light and the belowground response to microorganisms are interconnected along the microbiota–root–shoot axis.

Here, we show that microbial root commensals can alleviate *A. thaliana* growth deficiency under suboptimal light, and we observe a direct link between bacterial community composition and the prioritization of microbiota-induced growth over defence responses in shoots. We report that this microbiota- and light-dependent growth–defence trade-off requires the host transcriptional regulator MYC2. Our results imply that belowground responses to microorganisms and aboveground responses to light are integrated along a microbiota–root–shoot circuit to boost plant growth when the light condition is suboptimal.

## Results

### A synthetic root microbiota rescued *Arabidopsis* growth under low PAR

**Fig. 1:**
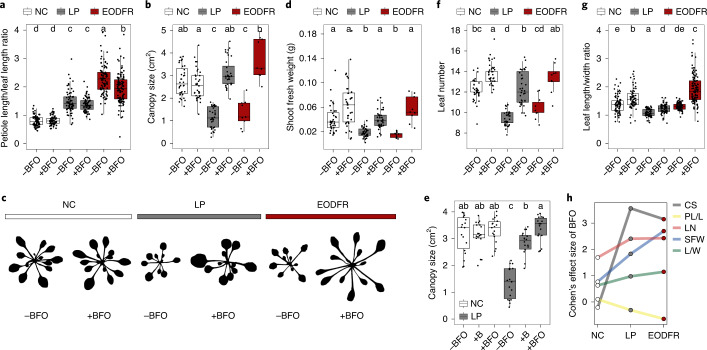
BFO-mediated modulation of leaf traits under LP and EODFR. **a**, Relative petiole length (petiole length divided by leaf length) of five-week-old *A. thaliana* grown in the FlowPot system in the absence (−) or presence (+) of a synthetic microbial community (BFO SynCom, B = 183 bacteria, F = 24 fungi, O = 7 oomycetes) under NC (white), LP (grey) or EODFR (dark red). There were three independent biological replicates (*n* = 489 leaves). **b**, Canopy size of five-week-old *A. thaliana* inoculated with and without the BFO SynCom under NC, LP and EODFR. There were three independent biological replicates (*n* = 162 plants). **c**, Representative images illustrating light- and BFO-induced changes in shoot morphology. **d**, Shoot fresh weight of five-week-old *A. thaliana* inoculated with and without the BFO SynCom under NC, LP and EODFR. There were three independent biological replicates (*n* = 162 plants) **e**, Canopy size of five-week-old *A. thaliana* grown in the FlowPot system in the absence (−) or presence (+) of a synthetic microbial community composed of either B or BFO under NC and LP. There were three independent biological replicates (*n* = 108 plants). **f**, Total leaf number of five-week-old *A. thaliana* inoculated with and without the BFO SynCom under NC, LP and EODFR. There were three independent biological replicates (*n* = 161 plants). **g**, Leaf length/width ratio of five-week-old *A. thaliana* inoculated with and without the BFO SynCom under NC, LP and EODFR. There were three independent biological replicates (*n* = 489 leaves). In **a**,**b**,**d**–**g**, the box plot boundaries reflect the interquartile range, the centre line is the median and the whiskers represent 1.5× the interquartile range from the lower and upper quartiles. The letters indicate statistical significance corresponding to Kruskal–Wallis with Dunn’s post hoc test (*α* = 0.05). **h**, Effect size of BFO on shoot morphological traits under NC, LP and EODFR. The different line colours reflect different shoot morphological traits. CS, canopy size; PL/L, petiole length/leaf length ratio; LN, leaf number; SFW, shoot fresh weight; L/W, leaf length/width ratio.

**Extended Data Fig. 1:**
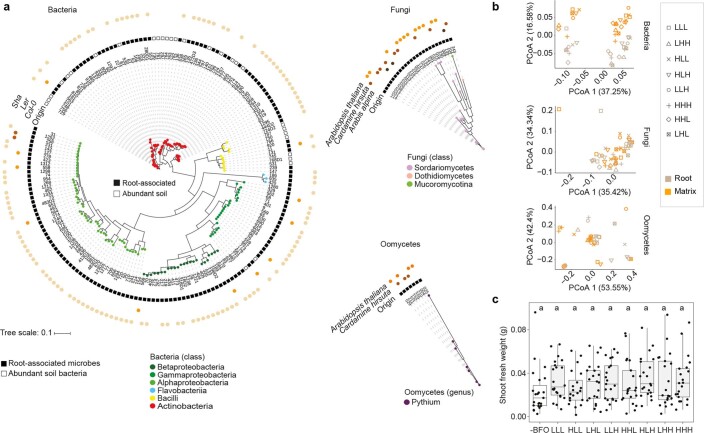
BFO SynCom composition, stability, and effect on plant growth. **a**, 183 Bacterial, 24 fungal, and 7 oomycete (BFO) strains were used to reconstitute a synthetic *A. thaliana* root microbiota. Taxonomic trees were constructed based on bacterial 16 s rRNA V5V7 sequences and fungal/oomycetal ITS1 sequences. The trees were edited in iTol^100^ and tracks represent (from the outside to the inside): name of the host plant/genotype (Shakdara: Sha, Landsberg erecta: Ler and Columbia-0: Col-0 are different *A. thaliana* accessions), origin of isolation (soil or root compartment), and microbial strain identification number. Circles at the extremity of the branches are colour coded according to strain taxonomic assignment. **b**, Impact of the relative abundance (RA) of B, F, and O on BFO community composition in roots and surrounding peat matrix in the FlowPot system. Data were visualized using a principal-component analysis (PcoA) based on Bray-Curtis dissimilarities. Samples are colour-coded according to the compartment, and BFO ratio conditions are depicted with different symbols. For inoculation, B, F, and O communities were assembled separately and microbial suspensions were adjusted to OD = 0.5 (2 ml/50 ml) for B and to 100 mg/50 ml for F and 40 mg/50 ml for O (high RA, **H**), or to OD = 0.5 (0.2 ml/50 ml) for B and to 10 mg/50 ml for F and 4 mg/50 ml for O (that is, 10 times dilution, low RA, **L**). Eight possible combinations of B-to-F-to-O RA ratios were then assembled and used to repopulate sterile peat in the FlowPot system. Three independent biological replicates (n = 51 samples). **c**, Shoot fresh weight (in g) of five-week-old *A. thaliana* inoculated with BFO at mixed at different B-to-F-to-O RA ratios or without BFO (-BFO). Three independent biological replicates (n = 191 plants). Letters indicate statistical significance corresponding to Kruskal-Wallis with Dunn’s *post hoc* test (α = 0.05). Boxplot boundaries reflect the inter-quartile range, the centre line is the median, and whiskers represent 1.5× interquartile range of lower and upper quartiles.

**Extended Data Fig. 2:**
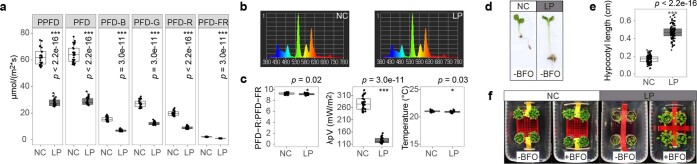
Difference in light conditions between LP and NC. **a**, Light parameters measured in the FlowPot system at different positions in the plant growth chamber (λp = 545 nm). PPFD: Photosynthetic Photon Flux Density defined in 400~700 nm, PFD: Photosynthetic Photon Flux Density defined in 380~780 nm, PFD-B: Photosynthetic Photon Flux Density in blue field (400~500 nm), PFD-G: Photosynthetic Photon Flux Density in green field (500~600 nm), PFD-R: Photosynthetic Photon Flux Density in red field (600~700 nm), PFD-FR: Photosynthetic Photon Flux Density in far-red field (700~780 nm). Asterisks indicate statistical significance (Mann-Whitney U test, *** *P* < 0.001). **b**, Spectrum in the FlowPot system under NC (normal light, left) and LP (low PAR, right). The y-axis is the quantified quantity of light (scaled to 0-1) automatically from the spectral PAR meter PG100N. **c**, R:FR ratio (left), λpV (Peak wavelength value: the highest power in the measured spectrum, middle), and temperature (right) measured in the FlowPot system at different positions in the plant growth chamber under NC and. Asterisks indicate statistical significance (T-test for R:FR ratio, Mann-Whitney U test for Peak wavelength value and temperature, *** *P* < 0.001, * *P* < 0.05). **d**, Representative pictures showing ten-day-old germ-free plants grown in the FlowPot system under NC and LP. **e**, Hypocotyl length (in cm) of ten-day-old germ-free *A. thaliana* grown in the FlowPot system under NC and LP. Three independent biological replicates (n = 240 plants). Statistical significance between LP and NC is indicated with asterisks (Mann-Whitney U test, *** *P* < 0.001). **f**, Representative pictures showing five-week-old plants growing in the FlowPot system under NC and LP in the absence (-) or presence (+) of the BFO SynCom. **a**, **c**, and **e**, Boxplot boundaries reflect the inter-quartile range, the centre line is the median, and whiskers represent 1.5× interquartile range of lower and upper quartiles.

**Extended Data Fig. 3:**
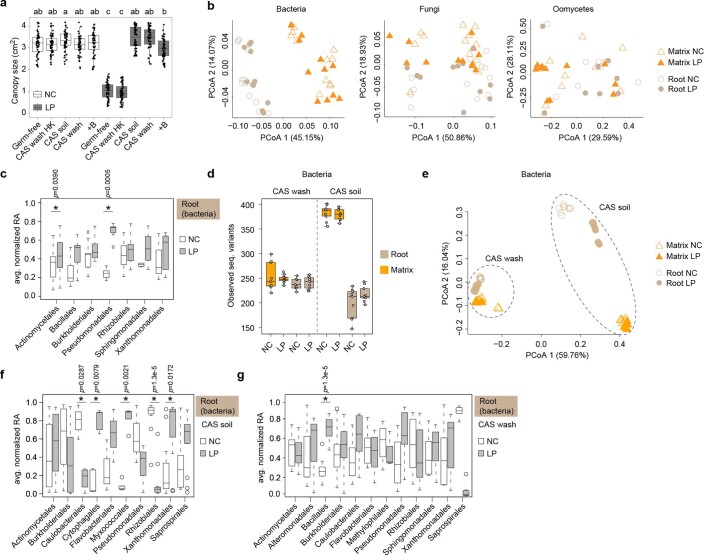
Influence of compartment and light conditions on BFO community composition. **a**, Canopy size of five-week-old *A. thaliana* grown under NC and LP in the FlowPot system in sterile peat (Germ-free), peat repopulated with a living or heat killed microbial wash from the Cologne Agricultural Soil (CAS wash or CAS wash HK) or with the bacterial SynCom (+B, 183 strains). Three independent biological replicates (n = 541 plants). Letters indicate statistical significance corresponding to Kruskal-Wallis with Dunn’s *post hoc* test (α = 0.05). **b**, Effect of “Compartment” and “Light” on B, F, and O in roots and matrix in the FlowPot system. Three independent biological replicates (B: n = 47 samples, F: n = 48, O: n = 37). **c**, Mean normalized RA in LP/NC for bacterial strain variants sorted by taxonomic order. Only strain variants that were consistently found across all samples (n = 24) were selected. Bacterial orders with less than two members were not considered (strain variants: Act. n = 28, Bac. n = 6, Bur. n = 9, Pse. n = 7, Rhi. n = 20, Sph. n = 4, Xan. n = 8). **d**, Observed bacterial sequence variants detected in roots and surrounding peat (CAS wash) or soil (CAS soil) matrix five weeks post inoculation in the FlowPot system. Three independent biological replicates (n = 72 samples). **e**, Bacterial community composition in roots and surrounding peat (CAS wash) or soil (CAS soil) matrix in the FlowPot system. **f** and **g**, Mean normalized RA in LP/NC for bacterial strain variants (sorted by taxonomic order) in roots of five-week-old plants grown in the CAS soil (**f**) or in peat repopulated with the CAS wash (**g**). Only strain variants consistently found across all samples were considered (n = 121/141, CAS soil/CAS soil washes). **c**, **d**, **f**, and **g**, Significant differences between norm. RA in LP and NC are shown (Mann-Whitney-U test, *P* < 0.05). **a**, **c**, **d**, **f**, and **g**, Boxplot boundaries reflect the inter-quartile range, the centre line is the median, and whiskers represent 1.5× interquartile range of lower and upper quartiles.

We hypothesized that a microbiota–root–shoot axis exists in plants, allowing them to take advantage of belowground microbial commensals to orchestrate aboveground stress responses. First, we assembled a synthetic microbial consortium (SynCom; Extended Data Fig. 1a and Supplementary Table 1) with phylogenetically diverse bacteria (B, 183 strains), fungi (F, 24 strains) and oomycetes (O, 7 strains) that naturally colonize the roots of healthy *A. thaliana*^12,29^. Eight versions of this BFO SynCom containing B, F and O communities mixed at different relative abundances (RAs) in the starting inoculum were used to recolonize the roots of germ-free *A. thaliana* in the gnotobiotic FlowPot system^30^. Sequencing of the 16S rRNA gene (B) and internal transcribed spacer (ITS) (F and O) confirmed that the initial difference in RA between the three microbial groups did not affect output root microbiota assembly five weeks post-inoculation, on the basis of principal component analysis (Extended Data Fig. 1b) and constrained analysis of principal coordinates (CAP) (B: *P* = 0.921; F: *P* = 0.476; O: *P* = 0.075). Similarly, differences in the B-to-F-to-O RA did not affect plant growth (Kruskal–Wallis, *P* = 0.226; Extended Data Fig. 1c). We then tested the relevance of this multikingdom microbial SynCom for plant growth under suboptimal light conditions. We manipulated light conditions in our plant growth chamber in two ways: (1) by restricting shoot exposure to direct light (low PAR (LP), reduction in light quantity; Extended Data Fig. 2a,b), resulting in a ~55% reduction in photosynthetic photon flux density (400–700 nm; normal light conditions (NC), 62.35; LP, 27.91; Mann–Whitney *U*-test, *P* < 2.2 × 10^−16^; Extended Data Fig. 2a) while only marginally affecting the ratio of red to far-red light (NC, 9.24; LP, 9.14; Extended Data Fig. 2c) and the temperature (NC, 21.02 °C; LP, 20.90 °C; Extended Data Fig. 2c) and (2) by exposing shoots to far-red light (740 nm) for 15 minutes at the end of the day (EODFR (ref. ^31^), reduction in light quality). Hypocotyl length (Extended Data Fig. 2d,e) and relative petiole length (petiole length divided by corresponding leaf length; LP: 1.9-fold, *P* < 2.2 × 10^−16^; EODFR: 2.8-fold, *P* < 2.2 × 10^−16^; Mann–Whitney *U*-test; Fig. 1a) were increased under suboptimal light conditions, consistent with stereotypical shade phenotypes previously described^32–34^. Compared with five-week-old plants grown under NC, those exposed to LP and EODFR showed a significant reduction in canopy size (LP: 2.4-fold, *P* = 5.45 × 10^−13^; EODFR: 2.1-fold, *P* = 2.88 × 10^−4^; Mann–Whitney *U*-test; Fig. 1b,c and Extended Data Fig. 2f) and shoot fresh weight (LP: 2.2-fold, *P* = 2.32 × 10^−8^; EODFR: 2.2-fold, *P* = 4.11 × 10^−5^; Mann–Whitney *U*-test; Fig. 1d) in the absence of the BFO SynCom. Remarkably, the presence of BFO rescued LP- and EODFR-mediated decreases in canopy size and shoot fresh weight to control levels (Fig. 1b–d). Furthermore, recolonization experiments with B in the presence or absence of F and O communities indicated that B commensals alone partially rescued plant growth under LP, whereas the synergistic contribution of B and filamentous eukaryotes was needed for the full rescue (Kruskal–Wallis with Dunn’s post hoc test, *P* < 0.05; Fig. 1e). Experiments with a natural soil (Cologne agricultural soil (CAS)) and with peat repopulated with a microbial wash from CAS validated the robustness of the plant growth rescue under LP, irrespective of microbial diversity and soil conditions (Extended Data Fig. 3a and Supplementary Table 1). The presence of BFO also led to a significant increase in leaf number (Fig. 1f) and the leaf length/width ratio (a proxy of leaf shape; Fig. 1g) (Kruskal–Wallis with Dunn’s post hoc test, *P* < 0.05). Inspection of the magnitude of BFO-mediated modification of leaf traits indicated quantitative differences among the tested parameters and revealed that the amplitude of the BFO effect was always greater under LP and EODFR than under the control light condition (Cohen’s effect size; Fig. 1h). Our results indicate that belowground microbial commensals promote the plant’s ability to grow under suboptimal light conditions by promoting canopy size and to a lesser extent by modulating canopy shape.

### Aboveground light conditions modulate root microbiota assembly

**Fig. 2:**
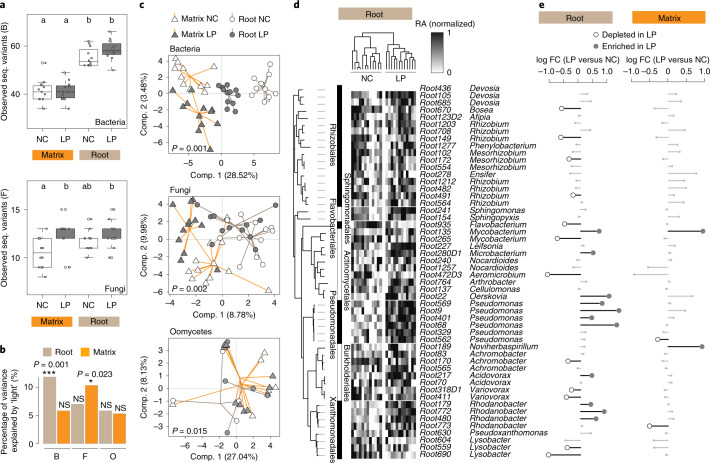
LP-mediated modulation of root microbiota assembly. **a**, Number of B (top) and F (bottom) strain variants detected in roots and the peat matrix five weeks after BFO inoculation in the FlowPot system. The 183 B, 24 F and 7 O strains were grouped into 115 B, 24 F and 7 O strain variants discriminated at single-nucleotide resolution against reference 16S rRNA and ITS sequences. There were three independent biological replicates (*n* = 48 samples). The letters indicate statistical significance corresponding to Tukey’s HSD (*α* = 0.05). Only samples with more than 1,000 reads were considered. The box plot boundaries reflect the interquartile range, the centre line is the median and the whiskers represent 1.5× the interquartile range from the lower and upper quartiles. **b**, Variation in B, F and O community composition explained by ‘light’ (NC versus LP) in root and matrix samples five weeks after BFO inoculation in the FlowPot system. CAP was used to quantify the percentage of variance explained by ‘light’ for each condition. ***P < 0.001; *P < 0.05; NS, not significant (CAP, ANOVA-like permutation test). **c**, Between-group variation in microbial community composition assessed using PLS-DA. Only samples with more than 1,000 reads were considered. The percentage of variance explained by the grouping factor is indicated along the *x* and *y* axes (B, *n* = 47 samples; F, *n* = 48; O, *n* = 26). Comp., component. **d**, Normalized RAs of prevalent B strain variants detected in roots across NC and LP. Sample-wise normalized RA was calculated for each strain variant and is depicted in the heat map next to the taxonomic tree. Only strain variants with an average RA ≥ 0.1% across samples were considered (*n* = 24 samples). The taxonomic tree was constructed on the basis of a maximum likelihood method using full-length 16S rRNA sequences. **e**, Sample-wise log fold change in RA measured for each prevalent strain variant between LP and NC in root and matrix samples. Differential RA with statistically significant *P* values is shown (edgeR, generalized linear model, *P* < 0.05).

**Extended Data Fig. 4:**
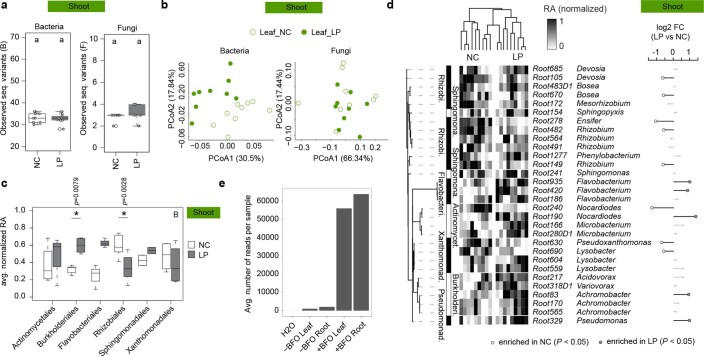
Influence of light conditions on leaf BFO community composition. **a**, Number of bacterial (B, left) and fungal (F, right) strain variants detected in plant shoots. Three independent biological replicates (n = 18 samples). Letters indicate statistical significance corresponding to Tukey´s HSD (α = 0.05). NC: normal light condition, LP: low photosynthetically active radiation. **b**, Effect of “Compartment” and “Light” on B and F community composition in shoots in the FlowPot system visualized by PCoA (Upper panel: B, Lower panel: F, Empty circles = NC, filled circles LP). Three independent biological replicates (B: n = 18 samples, F: n = 18)**. c**, Mean normalized RA in LP/NC for bacterial strain variants detected in plant shoots sorted by taxonomic order. Only strain variants that were consistently found across all samples (n = 18) were selected. Bacterial orders with less than two members were not considered (strain variants: Act. n = 4, Bur. n = 5, Fla. n = 3, Rhi. n = 10, Sph. n = 2, Xan. n = 4). Significant differences between norm. RA in LP and NC are shown (Mann-Whitney-U test, *P* < 0.05). **d**, Normalized RA of prevalent B strain variants detected in shoots across NC and LP conditions. Sample-wise normalized RA was calculated for each strain variant and is depicted in the heatmap next to the taxonomic tree. Only strain variants with an average RA ≥ 0.1% across samples were considered (n = 18 samples). The taxonomic tree was constructed based on a maximum likelihood method using full length 16 S rRNA sequences. Sample-wise log2 fold-change in RA measured for each prevalent strain variant between LP and NC in shoot samples. Differential RA with statistically significant *P*-values is shown (edgeR, GLM, *P* < 0.05). **e**, Mean number of reads in water control samples used for DNA extraction (H_2_O), leaf and root samples of germ-free (-BFO Leaf, -BFO Root) and colonized plants (+BFO Leaf, +BFO Root). **a** and **c**, Boxplot boundaries reflect the inter-quartile range, the centre line is the median, and whiskers represent 1.5× interquartile range of lower and upper quartiles.

We tested whether changes in light conditions perceived by leaves can cascade along the shoot–root axis, thereby modulating root microbiota assembly. Using the same strategy described above, we monitored B, F and O community composition in roots and the surrounding peat matrix for both LP and NC using amplicon sequencing (Methods). The 183 B, 24 F and 7 O could be discriminated into 115, 24 and 7 strain variants, respectively, at single-nucleotide resolution against reference 16S rRNA and ITS sequences (Supplementary Table 1). Inspection of B, F and O strain variants detected across conditions indicated a weak or non-significant effect of the light condition on microbial alpha diversity (analysis of variance (ANOVA) followed by post hoc Tukey’s honestly significant difference (HSD); Fig. 2a). Principal coordinate analysis (PCoA) of Bray–Curtis dissimilarities (Extended Data Fig. 3b), combined with permutational multivariate ANOVA (PERMANOVA, Supplementary Table 2), revealed that the factor ‘compartment’ explained a significant part of the variance in B and F but not O community composition in our gnotobiotic system (‘compartment: root/matrix’; B: *R*^2^ = 0.40, *P* < 0.001; F: *R*^2^ = 0.13, *P* < 0.001; O: *R*^2^ = 0.02, *P* = 0.439; Supplementary Table 2). Overall, the factor ‘light’ did not affect F and O community composition but did significantly shape B community composition (‘light’, *R*^2^ = 0.07, *P* = 0.002; Supplementary Table 2). CAP analysis constrained by ‘light’ and conducted independently for root and matrix samples indicated a significant effect of the light condition on B community composition in roots (‘light’, 11.9%, *P* < 0.001) but not in surrounding peat samples (‘light’, *P* = 0.606, Fig. 2b). In contrast, ‘light’ had no significant effect on F and O community composition in roots (‘light’, F: *P* = 0.105, O: *P* = 0.574) and a weak impact on F community in the matrix (‘light’, F: *P* = 0.023, O: *P* = 0.659; Fig. 2b). Partial least squares-discriminant analysis (PLS-DA; Fig. 2c) together with PERMANOVA conducted on root and matrix samples separately (Supplementary Table 2) further validated the prominent effect of the light condition on the composition of root-associated B communities (‘light’; root, *R*^2^ = 0.23, *P* = 0.006; matrix, *R*^2^ = 0.05, *P* = 0.3) but not F and O communities (Supplementary Table 2). Pairwise-enrichment tests conducted between LP and NC conditions revealed a significant impact of the light condition on the RAs of 23 strain variants in root samples and only 4 in matrix samples (edgeR, generalized linear model, *P* < 0.05; Fig. 2d,e). Multiple *Pseudomonas* and *Rhodanobacter* isolates were specifically enriched in the root compartment between LP and NC (Fig. 2e and Extended Data Fig. 3c). Notably, LP-induced belowground shift in bacterial community was retained in the roots of *A. thaliana* grown in CAS or in peat repopulated with the CAS wash (CAS wash: ‘light’, 13.2%, *P* = 0.002; CAS soil: ‘light’, 24.6%, *P* = 0.001) (Extended Data Fig. 3d,e), and the aforementioned taxonomic changes between LP and NC were quantitatively similar in the context of the CAS wash but not when the natural soil was used (Extended Data Fig. 3c,f,g). Given the possible ectopic leaf colonization by root microbiota members in our gnotobiotic system^35^, we also tested whether BFO root commensals can be detected in leaves and the extent to which their RAs can be modulated by light conditions. Diversity analysis (Extended Data Fig. 4a), PCoA (Extended Data Fig. 4b) and enrichment tests (Extended Data Fig. 4c,d) revealed that 44%, 15% and 0% of B, F and O strain variants detected belowground were colonizing aboveground leaf tissues, and that the composition of ectopic B assemblages was also modulated by light conditions, with 13 strain variants showing light-dependent differential growth (Extended Data Fig. 4d). Sequencing of blank controls from DNA extractions and control samples from germ-free plants confirmed the sterility of our system and validated our sequencing-based detection method (Extended Data Fig. 4e). Our results indicate that shoot exposure to suboptimal light triggers host-dependent modulation of the growth of root-associated and ectopic leaf-associated bacterial taxa in a complex multikingdom synthetic microbiome.

### Host prioritizes microbiota-induced growth over defence under LP

**Fig. 3:**
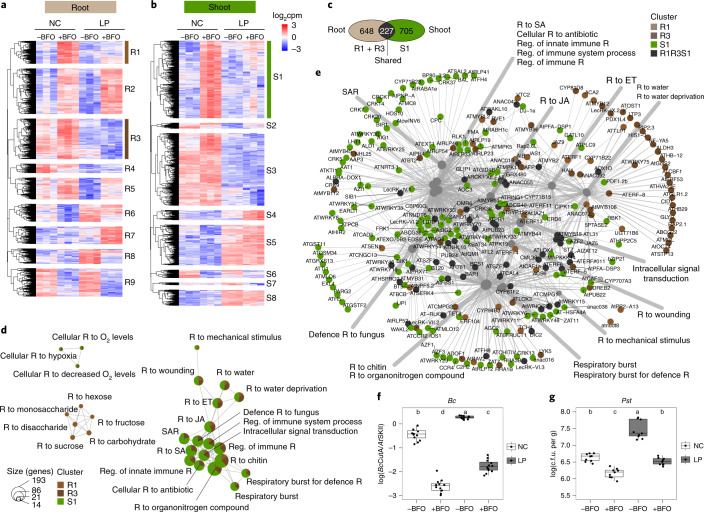
Light- and BFO-mediated transcriptional reprogramming in roots and shoots. **a**,**b**, Transcript profiling of 3,013 *A. thaliana* genes significantly regulated in root samples (**a**) and transcript profiling of 2,790 *A. thaliana* genes significantly regulated in shoot samples (**b**) (|log_2_FC| ≥ 1, empirical Bayes statistics, FDR < 0.05) based on all pairwise sample comparisons. The gene set was split into nine gene expression clusters in roots (R1 to R9) and eight gene expression clusters in shoots (S1 to S8). There were three independent biological replicates (*n* = 24 samples). **c**, Number of genes shared between root clusters R1 and R3 and shoot cluster S1 or specific to each of the two groups. **d**, GO term enrichment network depicting the top 12 most significantly enriched GO terms (hypergeometric test with Bonferroni correction, *P* < 0.05) detected in clusters R1, R3 and S1. Each GO term is represented as a circle, and the contribution of each cluster to the overall GO term enrichment is shown. The size of the GO term reflects the number of genes enriched in the GO term. R, response; reg., regulation; ET, ethylene. **e**, Gene-concept network depicting linkages between genes and the associated top 12 most significantly enriched GO terms detected in clusters R1, R3 and S1. Each node represents a gene and is colour-coded according to the different clusters. **f**, qPCR-based quantification of *Bc* growth in *A. thaliana* leaves five days after pathogen droplet inoculation in the FlowPot system. There were four independent biological replicates (*n* = 48 plants). **g**, Colony-count-based quantification of *Pst* growth in *A. thaliana* leaves five days after pathogen spray inoculation in the FlowPot system. There were three independent biological replicates (*n* = 36 plants). In **f** and **g**, the box plot boundaries reflect the interquartile range, the centre line is the median and the whiskers represent 1.5× the interquartile range from the lower and upper quartiles. The letters indicate statistical significance (one-way ANOVA followed by post hoc Tukey’s HSD, *α* = 0.05). cpm, counts per million; c.f.u., colony-forming units.

**Extended Data Fig. 5:**
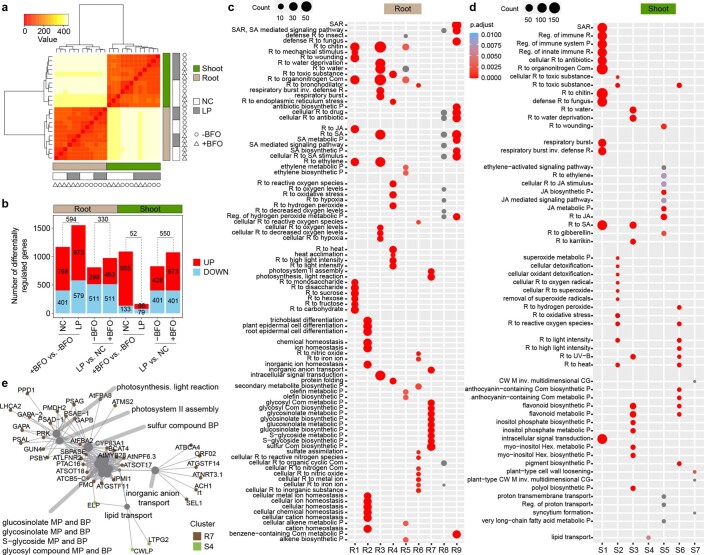
Transcriptional reprogramming in shoot and root in response to light and BFO. **a**, Heatmap showing the hierarchical relationship between samples used for RNAseq. Three independent biological replicates (n = 24 samples). NC: normal light condition, LP: low PAR. -BFO: no microbes (that is, germ-free), +BFO: with microbes. B: bacteria, F: fungi, O: oomycetes. The colour key on the top left side stands for sample distances (using “*dist*” function in “DESeq” R package to the transpose of the transformed count matrix to get sample-to-sample distances) corresponding to the colour range in the heatmap. **b**, Bar plot showing the numbers of differentially regulated genes among all possible pairwise comparisons. Red colour and blue colour filled bars represent significantly up- and down-regulated genes between the corresponding comparisons, respectively. The numbers highlighted inside the bars indicate the total number of up- or down- regulated genes between the corresponding comparisons. The numbers above the bars represent the numbers of genes shared between the corresponding conditions. **c**, and **d**, Gene Ontology (GO) term enrichment depicting the top 12 most significantly enriched GO terms (Hypergeometric test with Bonferroni correction, *P* < 0.05) detected in all clusters for root samples and shoot samples. The size of point reflects the amount of gene numbers enriched in this GO term. The colour of point means the p value ((Hypergeometric test with Bonferroni correction). R: response, Reg. Regulation, ET: ethylene, SA: salicylic acid, JA: jasmonic acid, SAR: systemic acquired resistance, CW: cell wall, CG: cell growth, P: process, Com: compound, Hex: hexakisphosphate, inv.: involved in. **e**, Gene-concept network (cnetplot function in R) depicting linkages between genes and associated top 12 most significantly enriched GO terms detected in clusters R7 (root) and S4 (shoot). Each node represents a gene and is colour-coded according the different cluster names. MP: metabolic process. BP: biosynthetic process.

We hypothesized that plant responses to BFO commensals and light are interconnected, thereby orchestrating resource investment into shoots under LP. We profiled the root and shoot transcriptomes of BFO-colonized and germ-free *A. thaliana* exposed to LP and NC in the gnotobiotic FlowPot system five weeks after BFO inoculation (Supplementary Table 3). PERMANOVA (Supplementary Table 3) and pairwise correlations among samples (Extended Data Fig. 5a) indicated that the presence/absence of the BFO SynCom explained transcriptome differentiation in root samples more than the light condition (microorganisms: *R*^2^ = 0.361, *P* < 0.001; light: *R*^2^ = 0.262, *P* < 0.001; Supplementary Table 3), whereas differentiation in the shoot transcriptome showed the opposite pattern (microorganisms: *R*^2^ = 0.224, *P* = 0.003; light: *R*^2^ = 0.293, *P* < 0.001; Supplementary Table 3). Consistent with a microbiota–root–shoot axis, the aboveground shoot transcriptome was influenced by BFO commensals (shoot: ‘microorganisms: light’, *R*^2^ = 0.091, *P* = 0.041), while the belowground root transcriptome was modulated by the light condition (root: ‘microorganisms: light’, *R*^2^ = 0.118, *P* = 0.038) (Supplementary Table 3). Hierarchical clustering of the expression profiles of all genes identified as differentially regulated across conditions (|log_2_fold change (FC)| ≥ 1; empirical Bayes statistics; false discovery rate (FDR), <0.05; Extended Data Fig. 5b) identified nine gene expression clusters in root samples (R1–R9, *n* = 3,013 genes; Fig. 3a) and eight in shoot samples (S1–S8, *n* = 2,790 genes; Fig. 3b). The presence of the BFO SynCom triggered light-independent upregulation of genes involved in ion homeostasis (root, R2) and cell differentiation (root, R2), as well as downregulation of genes involved in the response to salicylic acid (SA) (root, R9) and anthocyanin biosynthesis (shoot, S6) (Fig. 3a,b, Extended Data Fig. 5c,d and Supplementary Table 3). In contrast, LP triggered BFO-independent upregulation of genes involved in the response to jasmonic acid (JA) and gibberellin (GA) (shoot, S5) as well as downregulation of genes involved in the flavonoid metabolic process (shoot, S3) and response to high light intensity (root, R4) (Fig. 3a,b, Extended Data Fig. 5c,d and Supplementary Table 3). Furthermore, we identified clusters for which gene expression was modulated by both light and BFO conditions (R1, R3, R7, R8, S1 and S4), probably explaining BFO-mediated host rescue in LP (Fig. 3a,b). Particularly, genes belonging to clusters R1, R3 and S1 were upregulated in the presence of the BFO SynCom under NC but not under LP (Fig. 3a,b). A substantial fraction of the genes (227) did overlap between root clusters R1 and R3 and the shoot cluster S1 (Fig. 3c), and Gene Ontology (GO) term analysis revealed consistent enrichments of immune-related processes, including the response to chitin; the response to SA, JA and ethylene; the response to organonitrogen compounds; and the regulation of immune responses, among others (hypergeometric test with Bonferroni correction, *P* < 0.05; Fig. 3d, Extended Data Fig. 5c,d and Supplementary Table 3). Transcripts with conserved expression patterns in roots and shoots primarily encode transcription factors (WRKY33, WRKY40, MYB15, MYB51, ANAC042 and ANAC055), receptor-like protein/kinase (WAKL10, RLK3 and CRK9), ethylene-responsive elements (ERF6 and ERF11) or calmodulin binding proteins (CBP60G and CML38) (Fig. 3e). Immune response activation in response to the BFO SynCom under NC was more extensive aboveground than belowground and involved several well-known immune-related genes that act through multiple pathways (Fig. 3e). These genes encode transcription factors, proteins and enzymes involved in indole glucosinolate biosynthesis (MYB51 and CYP81F2), camalexin production (CYP71B15), SA response (PR1, BGL2, FRK1, EDS5 and PAD4), systemic acquired resistance (SAR) (SARD1, ALD1, AZI1 and DOX1) and to a lesser extent ethylene/JA responses (PDF1.2b) (Fig. 3e). The absence of transcriptional upregulation of these BFO-induced immune responses under LP (S1, R1 and R3) was accompanied by transcriptional upregulation of genes involved in gluconeogenesis in roots and lipid transport in leaves (R7 and S4; Extended Data Fig. 5e and Supplementary Table 3). Our results demonstrate that *A. thaliana* defence responses induced by multikingdom microbial commensals are modulated by light, probably contributing to the prioritization of microbiota-induced growth over defence under LP.

### Light and root microbiota modulate leaf pathogen resistance

We hypothesized that aboveground defence triggered by BFO is weakened under LP compared with NC. We tested this hypothesis in our gnotobiotic plant system by inoculating leaves of four-week-old *A. thaliana* (NC − BFO, NC + BFO, LP − BFO and LP + BFO) with the necrotrophic fungal pathogen *Botrytis cinerea* B05.10 (*Bc*, droplet inoculation, 1 × 10^3^ spores) or the hemibiotrophic bacterial pathogen *Pseudomonas syringae* pv. *tomato* DC3000 (*Pst*, spray inoculation, OD = 0.2). The evaluation of pathogen growth in planta by quantitative PCR (qPCR) (*Bc*, Fig. 3f) or colony counting (*Pst*, Fig. 3g) revealed major influences of both light and SynCom conditions on disease resistance five days after pathogen inoculation (ANOVA followed by post hoc Tukey’s HSD, *P* < 0.05; Fig. 3f,g). Plants colonized by the BFO SynCom under NC were the most resistant to both leaf pathogens, which is consistent with extensive BFO-triggered immune responses observed in the RNA-seq data and the presence of putatively protective commensals that ectopically colonize aboveground shoot organs^36^ (Fig. 3b,f,g). In contrast, germ-free plants facing LP were the most susceptible to both pathogens and failed to mount effective immune responses. Although the amplitude of BFO-induced leaf pathogen protection (versus germ-free control) was quantitively similar in NC and LP, plants were more susceptible when the light condition was suboptimal (ANOVA followed by post hoc Tukey’s HSD, *P* < 0.05; Fig. 3f,g). Despite the transcriptional shut-down of immune responses observed in LP + BFO versus NC + BFO conditions, and despite massive investment into growth, colonized plants grown under LP remained largely able to resist aboveground pathogens (Figs. 1b and 3f,g). Taken together, canopy size data, pathogen inoculation assays and RNA-seq experiments suggest that sterile plants failed to grow and defend under LP, whereas colonized plants can grow and resist but probably prioritize growth over defence responses to overcome the light limitation stress.

### Microbiota-mediated plant rescue depends on several host pathways

**Fig. 4:**
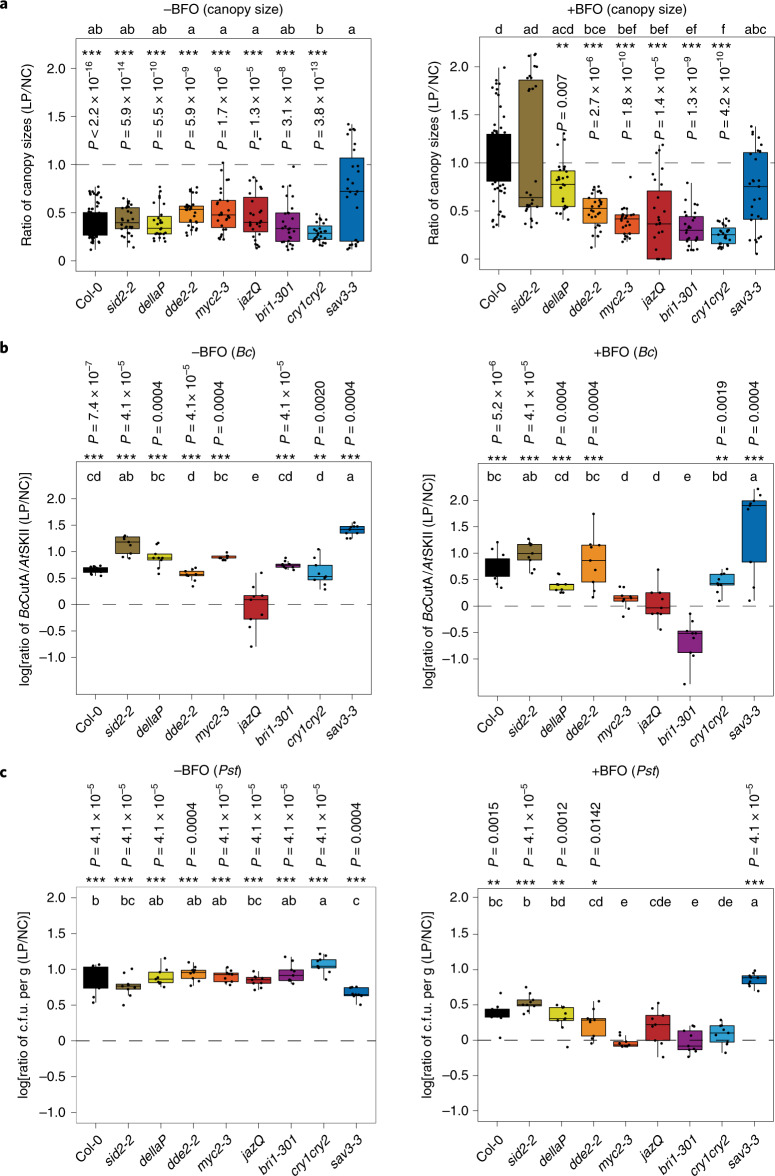
BFO-induced plant growth promotion in LP requires multiple host pathways. **a**, Differential canopy size between LP and NC for *A. thaliana* Col-0 and eight mutants grown in the FlowPot system under germ-free conditions (−BFO, left) or in the presence of the BFO SynCom (+BFO, right). The ratio of canopy sizes (five-week-old plants) was computed between LP and NC across three independent biological replicates (*n* = 1,188, −BFO and +BFO samples). Statistical significance across genotypes is indicated with letters (Kruskal–Wallis with Dunn’s post hoc test, *α* = 0.05). Statistical significance between LP and NC is indicated with asterisks (Mann–Whitney *U*-test, ***P < 0.001, **P < 0.01, *P < 0.05). **b**, Differential *Bc* growth between LP and NC in leaves of *A. thaliana* Col-0 and eight mutants grown in the FlowPot system (left, −BFO; right, +BFO). *Bc* growth was quantified by qPCR in *A. thaliana* leaves five days after pathogen droplet inoculation. The ratio of *Bc* loads was computed between LP and NC across three independent biological replicates for each genotype (*n* = 336, −BFO and +BFO samples). **c**, Differential *Pst* growth between LP and NC in leaves of *A. thaliana* Col-0 and eight mutants grown in the FlowPot system (left, −BFO; right, +BFO). *Pst* growth was quantified by colony counting in *A. thaliana* leaves five days after pathogen spray inoculation. The ratio of *Pst* loads was computed between LP and NC across three independent biological replicates for each genotype (*n* = 324, −BFO and +BFO samples). In **b** and **c**, statistical significance across genotypes is indicated with letters (one-way ANOVA followed by post hoc Tukey’s HSD, *α* = 0.05). Statistical significance between LP and NC is indicated with asterisks (Mann–Whitney *U*-test, ***P < 0.001, **P < 0.01, *P < 0.05). In **a**–**c**, the box plot boundaries reflect the interquartile range, the centre line is the median and the whiskers represent 1.5× the interquartile range from the lower and upper quartiles.

**Extended Data Fig. 6:**
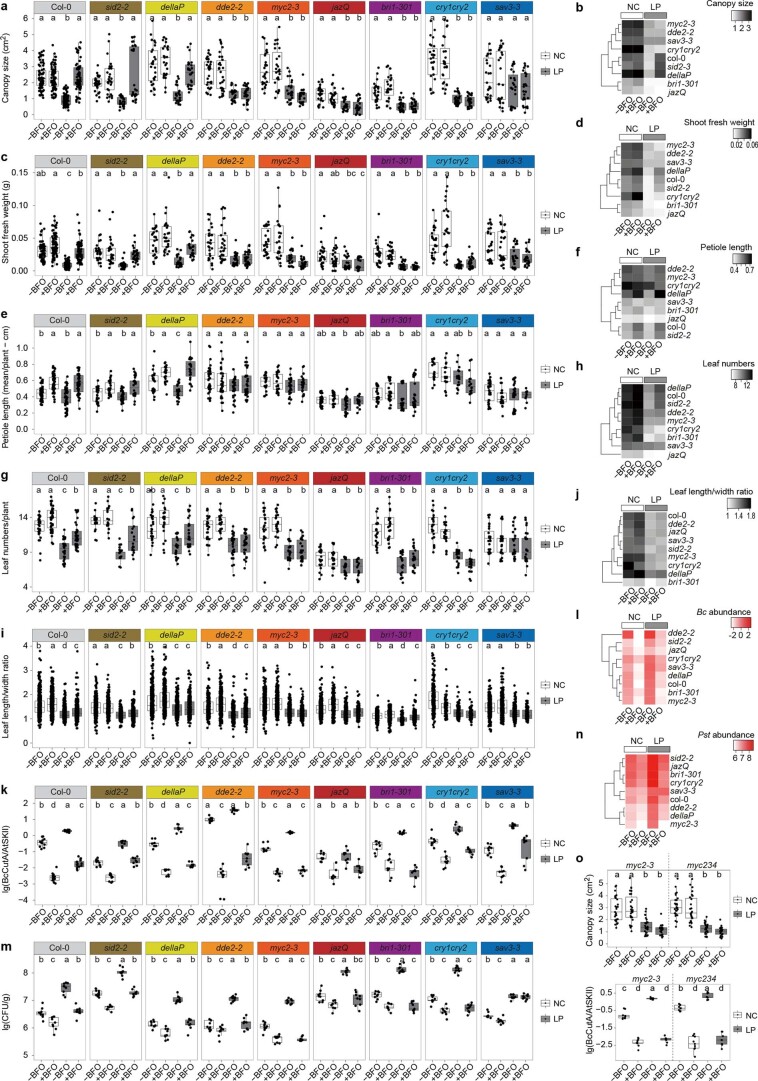
BFO-induced growth over defence in LP depends on JA and BR pathways. Canopy size (**a**), shoot fresh weight (**c**), mean petiole length (**e**), total leaf number (**g**), leaf length/width ratio (**i**) of five-week-old *A. thaliana* plants grown under NC and LP, in the absence (-BFO) or presence of the BFO SynCom (+BFO). Three independent biological replicates are shown (**a**: n = 1188 plants; **c**: n = 1188 plants; **e**: n = 1095 plants; **g**: n = 925 plants; **i**: n = 9,708 leaves). Letters indicate statistical significance (Kruskal-Wallis with Dunn’s *post hoc* test, α = 0.05). Summary heatmap depicting the mean of canopy size (**b**), shoot fresh weight (**d**), mean petiole length per plant (**f**), total leaf number per plant (**h**), leaf length/width ratio (**j**) measured across plant genotypes and conditions. *B. cinerea* B05.10 (*Bc*) growth (**k**) and *Pseudomonas syringae pv. tomato* DC3000 (*Pst*) growth (**m**) in leaves of *A. thaliana* Col-0 and eight mutants grown under NC and LP, in the absence (-BFO) or presence of the BFO SynCom (+BFO), in the FlowPot system. Three independent biological replicates are shown (**k**: n = 336; **m**: n = 324). **k** and **m**, Statistical significance across conditions for each genotype is indicated with letters (one-way ANOVA followed by *post hoc* Tukey´s HSD, α = 0.05). Summary heatmap depicting relative *Bc* growth (**l**) and *Pst* growth (**n**) measured across plant genotypes and conditions. **o**, Canopy size (top) and *B. cinerea* B05.10 (*Bc*) growth (bottom) in leaves of five-week-old *myc2-3* and *myc234* mutants grown under NC and LP, in the absence (-BFO) or presence of the BFO SynCom (+BFO), in the FlowPot system. Three independent biological replicates are shown (Canopy size: n = 216; *Bc* growth: n = 72). Letters indicate statistical significance (Canopy size: Kruskal-Wallis with Dunn’s *post hoc* test, α = 0.05; *Bc* growth: one -way ANOVA followed by *post hoc* Tukey´s HSD, α = 0.05). **a, c, e, g, i, k, m**, Boxplot boundaries reflect the inter-quartile range, the centre line is the median, and whiskers represent 1.5× interquartile range of lower and upper quartiles.

BFO-mediated shoot growth promotion under LP probably results from complex microbiota–root–shoot signalling mechanisms involving light perception, plant development and immune responses. To identify host components required for the prioritization of microbiota-induced growth over defence under LP, we monitored plant growth as well as *Bc* and *Pst* colonization across several mutant plants in our gnotobiotic system (Extended Data Fig. 6). These plants included mutants impaired in SA and JA biosynthesis (SA, *sid2-2*; JA, *dde2-2*)^37,38^, JA and GA signalling (JA, *myc2-3/jin1-8* and *jazQ*; GA, *dellaP*)^24,39,40^, brassinosteroid signal transduction (*bri1-301*)^41^, light perception (*cry1* *cry2*)^42^ and indole-3-pyruvic-acid-dependent auxin biosynthesis (*sav3-3*)^43^ (Supplementary Table 4). Consistent with previous work^24,41,44–46^, we observed mutant versus Col-0 wild-type (WT) plant variation in vegetative growth under control conditions (NC − BFO), with enhanced growth for *dellaP* and *cry1* *cry2* mutants and reduced growth for *jazQ* and *bri1-301* mutants (Extended Data Fig. 6a–j,o; Mann–Whitney *U*-test, *P* < 0.05; statistical analysis not depicted). Irrespective of these differences in growth rate, LP-mediated reduction in canopy size was quantitatively similar across all mutants tested in the absence of the BFO SynCom (0.48-fold to 0.71-fold decrease in canopy size), except for the *sav3-3* mutant, which retained high growth under LP (Kruskal–Wallis with Dunn’s post hoc test; Fig. 4a). The similarity in Col-0 canopy size observed between LP and NC in the presence of BFO (+BFO; ratio of LP to NC close to 1; Fig. 4a) was largely retained in the *sid2-2*, *dellaP* and *sav3-3* mutants, indicating that the corresponding genes are dispensable for BFO-mediated canopy size enlargement under LP (Fig. 4a and Extended Data Fig. 6a,b). In contrast, canopy size was dramatically reduced under LP compared with NC for *dde2-2*, *myc2-3*, *jazQ*, *bri1-301* and *cry1* *cry2* mutants (+BFO; ratio of LP to NC close to 0.5; Mann–Whitney *U*-test, *P* < 0.01; Fig. 4a and Extended Data Fig. 6a,b), indicating that JA biosynthesis/signalling, brassinosteroid signal transduction and cryptochromes are needed for BFO-induced growth under LP. Integration of the belowground response to microbiota and the aboveground response to light therefore involved multiple points of control along the root–shoot axis. In our gnotobiotic system (NC + BFO, NC − BFO, LP − BFO and LP + BFO), we then inoculated leaves of four-week-old *A. thaliana* Col-0 and mutant plants with *Bc* or *Pst*. Consistent with previous work^39,47,48^, we observed genotype-specific differences in *Bc* and *Pst* susceptibility phenotypes under control conditions (NC − BFO), with the *dde2-2* and *sid2-2* mutants being the most susceptible and resistant to *Bc*, respectively, and the *sid2-2* and *myc2-3* mutants being the most susceptible and resistant to *Pst*, respectively (Extended Data Fig. 6k–o). Under sterile conditions (−BFO), all the mutants tested except *jazQ* (*Bc* condition) showed a significant and consistent increase in *Bc* and *Pst* load in their leaves in LP compared with NC (*Bc*, 3.70-fold to 25.69-fold increase in susceptibility, Fig. 4b; *Pst*, 4.66-fold to 11.94-fold increase in susceptibility, Fig. 4c), validating that LP-mediated reduction in canopy size in the absence of root commensals was also associated with impaired resistance to unrelated leaf pathogens (Fig. 4b,c). In contrast, most of the mutants impaired in BFO-mediated growth rescue in LP remained as resistant to *Bc* and *Pst* (*myc2-3*, *jazQ* and *bri1-301*) or *Pst* only (*cry1* *cry2*) as under NC (Mann–Whitney *U*-test, *P* < 0.05). Our results underline the potential roles of MYC2 and BRI1 as regulatory nodes balancing investment into microbiota-induced growth at the expense of defence under LP.

### A link between differential canopy size and root microbiota composition under LP

**Fig. 5:**
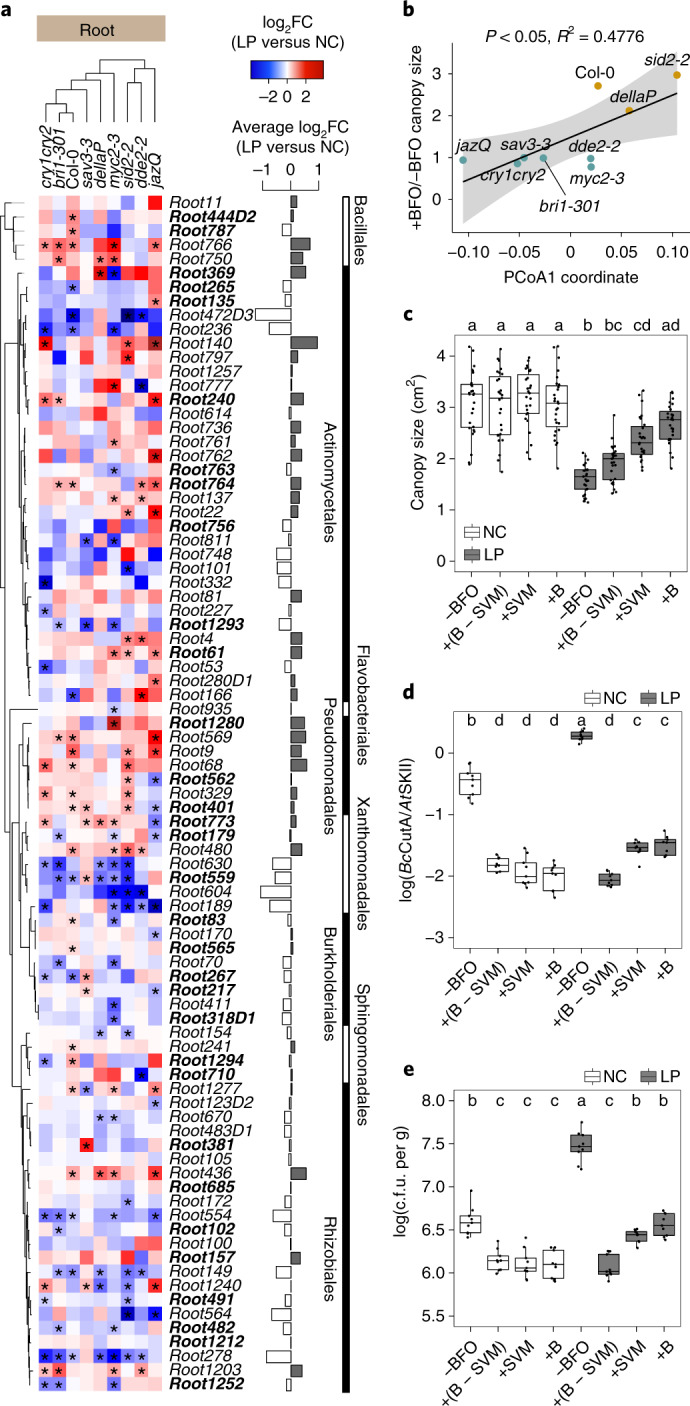
Link between B community composition and BFO-induced growth over defence in LP. **a**, Phylogeny-based heat map showing differential RA in roots under LP versus NC for each variant across all genotypes. Only strain variants (*n* = 85) consistently present across genotype samples were considered (number of samples per genotype, *n* = 9, except *jazQ* (*n* = 6) and Col-0 (*n* = 21)). Significant differences in RA between LP and NC are indicated by asterisks (edgeR, generalized linear model, *P* < 0.05). The bar plot next to the heat map represents the mean difference in RA between LP and NC across all genotypes. Strain variants highlighted in bold correspond to those identified through the SVM classifier. **b**, Linear regression between BFO-induced canopy size in LP (the canopy size under LP in +BFO divided by the mean value of the respective mutant under LP in −BFO) and bacterial community composition (the first axis of PCoA based on Bray–Curtis dissimilarities between samples). Values of *P* and *R*^2^ obtained with ANOVA are indicated in the figure. **c**, Canopy size of five-week-old *A. thaliana* grown in the FlowPot system in the absence (−BFO) or presence of synthetic bacterial communities under NC and LP. +B, all 183 bacterial strains; +SVM, 67 strains identified by the SVM approach as discriminant of the plant growth-rescue phenotype under LP; +(B − SVM), remaining 116 strains not predicted by the SVM approach. There were three independent biological replicates (*n* = 216 plants). The letters indicate statistical significance corresponding to Kruskal–Wallis with Dunn’s post hoc test (*α* = 0.05). **d**, qPCR-based quantification of *Bc* growth in *A. thaliana* leaves five days after pathogen droplet inoculation in the FlowPot system. **e**, Colony-count-based quantification of *Pst* growth in *A. thaliana* leaves five days after pathogen spray inoculation in the FlowPot system. In **d** and **e**, there were three independent biological replicates (*n* = 72 for each panel). The letters indicate statistical significance (one-way ANOVA followed by post hoc Tukey’s HSD, *α* = 0.05). In **c**–**e**, the box plot boundaries reflect the interquartile range, the centre line is the median and the whiskers represent 1.5× the interquartile range from the lower and upper quartiles.

**Extended Data Fig. 7:**
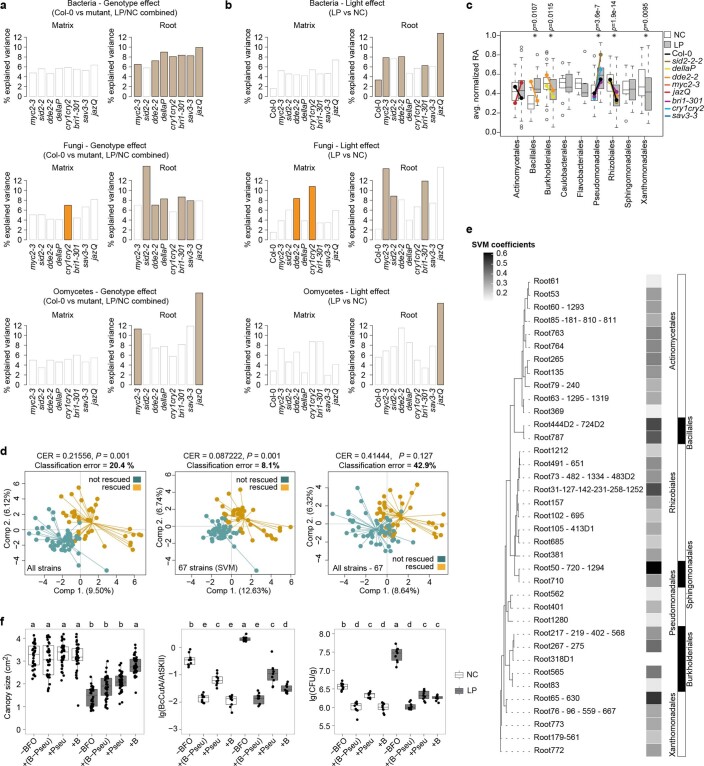
Genotype-dependent microbial community shifts between LP and NC. Genotype effect (**a**) and light effect (**b**) for all datasets (Bacteria upper panel, Fungi middle panel, Oomycetes lower panel). Significance is highlighted with coloured boxes (orange for matrix and brown for root, one-way ANOVA permutation test, *P* < 0.05). **c**, Mean normalized RA in LP/NC samples for bacterial strain variants for all genotypes, sorted by taxonomical order. Coloured dots depict means within genotypes that are significantly different (Mann-Whitney-U test, *P* < 0.05). **d**, Partial least square discriminant analysis (PLS-DA) testing the differentiation between rescued and non-rescued plant phenotypes in LP based on the composition of the bacterial communities across all mutants (183 strains, n = 90 samples, **left**), the subset of strains identified in the System Vector Machine (SVM) (67 strains, n = 90 samples, **middle**), and the bacterial strains without SVM strains (116 strains, n = 90 samples, **right**). Quality parameters of the analysis (P-value, CER and mean misclassification rate) are indicated in the figure. **e**, 16 S rRNA-based phylogeny of 67 strains that discriminate best BFO-induced plant phenotypes under LP conditions (that is, rescued *vs*. not rescued, SVM-RFE, R^2^ = 0.83). **f**, Canopy size (in cm^2^, **left**) of five-week-old *A. thaliana*, qPCR-based quantification of *Botrytis cinerea B05.10* (*Bc*) growth (**middle**), and Colony count-based quantification of *Pseudomonas syringae pv. tomato* DC3000 (*Pst*) growth (**right**) in *A. thaliana* leaves five days post pathogen inoculation in the FlowPot system in the absence (-) or presence (+) of a synthetic microbial community composed of either B without *Pseudomonas* strains (+(B-Pseu), 176 strains), or *Pseudomonas* strains only (+Pseu, 7 strains), or B ( + B, 183 strains) under NC (white) and LP (grey). Three independent biological replicates (Canopy size: n = 288; *Bc* growth: n = 72; *Pst* growth: n = 72). Letters indicate statistical significance (Canopy size: Kruskal-Wallis with Dunn’s *post hoc* test, α = 0.05; *Bc* and *Pst* growth: one-way ANOVA followed by *post hoc* Tukey´s HSD, α = 0.05). **c, f**, Boxplot boundaries reflect the inter-quartile range, the centre line is the median, and whiskers represent 1.5× interquartile range of lower and upper quartiles.

We asked whether differentiation in B community composition in the roots of WT plants was altered in the different mutants and could correlate with aboveground change in canopy size. We took advantage of the previous experiment in which the different mutants were grown in the gnotobiotic system under NC and LP to simultaneously monitor BFO assemblages in roots and the surrounding peat matrix five weeks after BFO inoculation. PERMANOVA confirmed the effect of the light condition on B community composition in the roots but not in the matrix samples (root, ‘light’: *R*^2^ = 0.014, *P* = 0.024) and revealed that B community differentiation was more extensively shaped by ‘genotype’ than by ‘light’ (root, ‘genotype’: *R*^2^ = 0.117, *P* < 0.001), which was validated by CAP (Supplementary Table 5 and Extended Data Fig. 7a). CAP analysis constrained by ‘light’ for each genotype indicated that light-mediated differentiation in B community composition was greater for *jazQ* (12.8%, *P* = 0.004), *dde2-2* (8.07%, *P* = 0.0265), *myc2-3* (7.87%, *P* = 0.005) and *bri1-301* (6.26%, *P* = 0.049) than for the WT (3.35%, *P* = 0.044) and was not significant for the other mutants (Extended Data Fig. 7b). Pairwise-enrichment tests conducted between LP and NC conditions for each genotype (edgeR, generalized linear model, *P* < 0.05; Fig. 5a) validated the increase in RA of root-associated Pseudomonadales and Actinomycetales observed in the WT, and revealed mutant-specific differences in abundance profiles at the strain (Fig. 5a) and class (Extended Data Fig. 7c) levels. To further test for potential associations between aboveground canopy phenotypes and belowground B community composition, we calculated canopy size variation for each mutant between LP − BFO and LP + BFO conditions and asked whether these quantitative differences were significantly linked to corresponding root microbiota composition under LP. Using a linear regression model, we observed a significant link between BFO-induced canopy growth across mutants and B community composition in roots (coordinate on PCoA axis 1, 46.9% of the variance in Bray–Curtis dissimilarities), which explained 47.7% of the variation in B community composition along PCoA1 (*R*^2^ = 0.4776, *F*_1,7_ = 8.314, *P* = 0.02353; Fig. 5b). We then divided the genotypes into two groups on the basis of the BFO-mediated growth induction (rescued: BFO-induced growth under LP; not rescued: lack of BFO-induced growth under LP) and validated that belowground change in B community composition can be used to discriminate those groups, with a mean classification error of 20.4% (PLS-DA, *P* < 0.001; Extended Data Fig. 7d). To identify the strain variants that allow the best discrimination of the two groups on the basis of variations in their RAs in LP, we trained a support vector machines (SVM) classifier with recursive feature elimination and identified a set of 37 strain variants that were sufficient to accurately predict the phenotype group (*R*^2^ = 0.83; Extended Data Fig. 7e and Supplementary Table 5). Using RA data of these 37 strain variants as an input for PLS-DA, we greatly improved the model quality (PLS-DA; *P* < 0.001; classification error, 8.1%; Extended Data Fig. 7d), whereas a similar analysis with the strain variants not selected by the SVM classifier (*n* = 68 strain variants) could no longer discriminate the two groups (PLS-DA; *P* > 0.05; classification error, 40%; Extended Data Fig. 7d). To further test the extent to which these SVM-defined strain variants (that is, 37, corresponding to 67 strains) drive investment in growth under LP, we performed recolonization experiments with either the full B SynCom (+B, 183 strains) or reduced SynComs containing or lacking the SVM-defined strains (+SVM, 67 strains; +(B − SVM), 116 strains; Fig. 5c–e). Remarkably, plants recolonized by the +SVM SynCom invested into growth at the expense of defence under LP, whereas those colonized by the +(B − SVM) SynCom failed to invest into growth and remained as resistant to *Bc* and *Pst* as under NC (Fig. 5c–e). A similar experiment with B SynComs lacking all seven *Pseudomonas* isolates (+(B − Pseu), 176 strains) or comprising only the *Pseudomonas* strains (+Pseu, 7 strains) further indicated that these commensals were necessary but not sufficient for driving investment into growth at the expense of defence (Extended Data Fig. 7f). Taken together, our results suggest that plant growth and defence phenotypes under LP are directly linked to belowground B community composition and that host modulation of belowground community assembly influences investment into growth over defence under LP.

### Priority to microbiota-induced growth over defence under LP requires MYC2

**Fig. 6:**
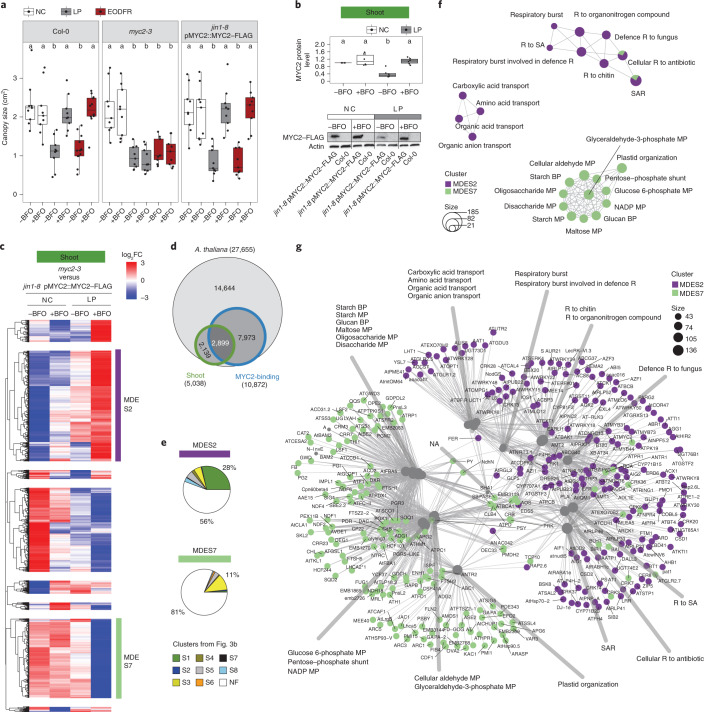
MYC2-dependent prioritization of BFO-induced growth over defence in LP. **a**, Canopy size of five-week-old WT (Col-0), mutant (*myc2-3*) and complemented (*jin1-8* pMYC2::MYC2–FLAG) *A. thaliana* grown in the FlowPot system in the absence (−) or presence (+) of the BFO SynCom under NC (white), LP (grey) or EODFR (dark red). There were three independent biological replicates (Col-0, *n* = 60 plants; *myc2-3*, *n* = 54 plants; *jin1-8* pMYC2::MYC2–FLAG, *n* = 54 plants). **b**, Quantified MYC2 protein abundance in shoots of five-week-old *jin1-8* pMYC2::MYC2–FLAG plants in the FlowPot system with or without BFO under either NC (white) or LP (grey). There were three independent biological replicates including six independent replicates of immunoblots (*n* = 48 samples). One immunoblot replicate is shown on the bottom (uncropped scan in Extended Data Fig. 9b). In **a** and **b**, the letters indicate statistical significance (Kruskal–Wallis with Dunn’s post hoc test, *α* = 0.05). The box plot boundaries reflect the interquartile range, the centre line is the median and the whiskers represent 1.5× the interquartile range from the lower and upper quartiles. **c**, Transcript profiling of 5,038 *A. thaliana* genes significantly regulated in shoots of the *myc2-3* mutant versus the *jin1-8* pMYC2::MYC2–FLAG line (|log_2_FC| ≥ 1, empirical Bayes statistics, FDR < 0.05). The gene set was split into eight major MDE gene expression clusters (MDES1 to MDES8). There were three independent biological replicates (*n* = 24 samples). **d**, Overlap between the number of MDE genes identified in shoots and all MYC2 target genes identified by ChipSeq in three independent studies^51–53^. **e**, Percentage of genes shared between MDES2 (top) and MDES7 (bottom) and clusters previously defined in Fig. 3b (S1 to S8). NF, not found in Fig. 3b. **f**, GO term enrichment network depicting the top 12 most significantly enriched GO terms (hypergeometric test with Bonferroni correction, *P* < 0.05) in MDES2 and MDES7. The size of the GO term reflects the number of genes enriched. R, response; BP, biosynthetic process; MP, metabolic process. **g**, Gene-concept network (cnetplot function in R) depicting linkages between genes and the associated top 12 most significantly enriched GO terms detected in clusters MDES2 and MDES7.

**Extended Data Fig. 8:**
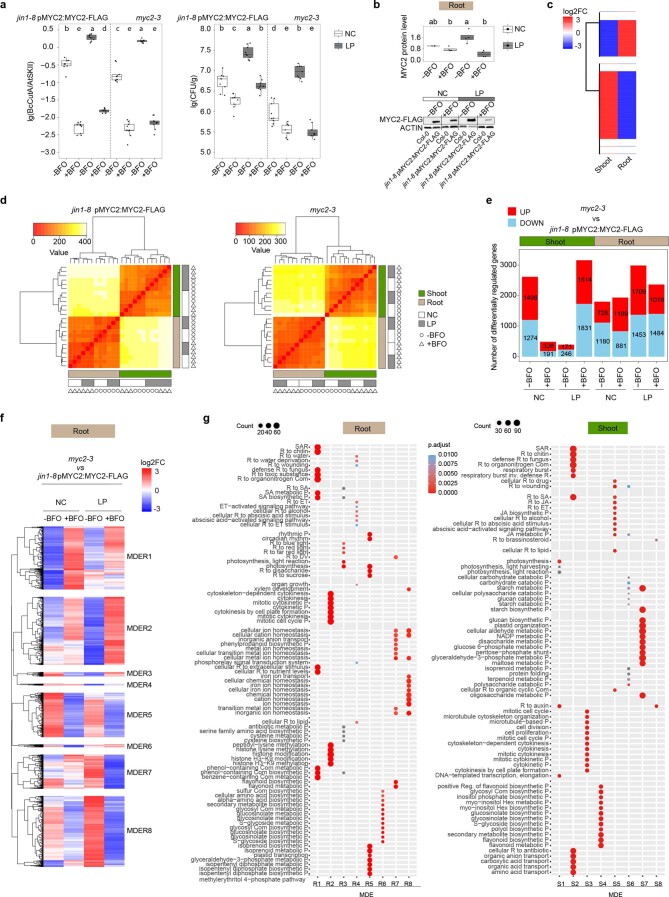
MYC2-dependent prioritization of BFO-induced growth over defence in LP. **a**, Pathogen growth (*Bc* growth, **left**; *Pst* growth, **right**) in *A. thaliana jin1-8* pMYC2:MYC2-FLAG line and *myc2-3* mutant leaves five-day-post pathogen inoculation. Three independent biological replicates (*Bc*: n = 72 plants; *Pst*: n = 72 plants). Letters indicate statistical significance (one-way ANOVA followed by *post hoc* Tukey´s HSD, α = 0.05). **b**, Quantified MYC2 protein abundance in roots of five-week-old *jin1-8* pMYC2:MYC2-FLAG line in the absence (-) or presence (+) of BFO under either NC (white) or LP (grey). Three independent biological replicates including four independent replicates of immunoblots (n = 32 samples). Letters indicate statistical significance (Kruskal-Wallis with Dunn’s *post hoc* test, α = 0.05). One immunoblots replicate was shown on the bottom. **a** and **b**, Boxplot boundaries reflect the inter-quartile range, the centre line is the median, and whiskers represent 1.5× interquartile range of lower and upper quartiles. **c**, The expression pattern of 571 shared genes between shoot and root significantly regulated by MYC2 in the presence of BFO under LP. **d**, Heatmaps showing the hierarchical relationship between samples from *jin1-8* pMYC2:MYC2-FLAG line (left) and *myc2-3* mutant (right) used for RNAseq. Three independent biological replicates (n = 48 samples). **e**, Bar plot showing the numbers of differentially regulated genes *myc2-3* mutant *vs. jin1-8* pMYC2:MYC2-FLAG line (|log2FC | ⩾ 1, Empirical Bayes Statistics, FDR < 0.05). **f**, Transcript profiling of 5,231 *A. thaliana* genes significantly regulated in root samples between *myc2-3* mutant and *jin1-8* pMYC2:MYC2-FLAG line (|log2FC | ⩾ 1, Empirical Bayes Statistics, FDR < 0.05). Three independent biological replicates (n = 24 samples). **g**, Gene Ontology (GO) term enrichment depicting the top 12 most significantly enriched GO terms (Hypergeometric test with Bonferroni correction, *P* < 0.05). The size of point reflects the amount of gene numbers enriched in this GO term. The colour of point means the p value (Hypergeometric test with Bonferroni correction). R: response, Reg. Regulation, ET: ethylene, SA: salicylic acid, JA: jasmonic acid, SAR: systemic acquired resistance, CW: cell wall, CG: cell growth, P: process, Com: compound, Hex: hexakisphosphate, inv.: involved in.

**Extended Data Fig. 9:**
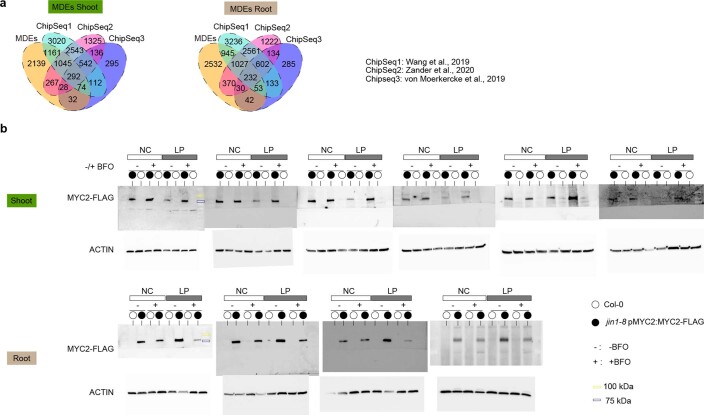
Overlap between MDEs genes and previously defined MYC2 target genes. **a**, Overlap between the number of MDE genes identified in shoot (**left**) or root (**right**) and all MYC2 target genes identified by ChipSeq experiments in three independent studies^51–53^. **b**, Immunoblots showing MYC2 protein abundance in shoot (**top**) and root (**bottom**) of five-week-old *jin1-8* pMYC2:MYC2-FLAG line grown in the FlowPot system in the absence (-) or presence (+) of the BFO SynCom under NC and LP. Three independent biological replicates (six independent replicates of shoot samples, n = 48 samples; four independent replicates of root samples, n = 32 samples).

**Extended Data Fig. 10:**
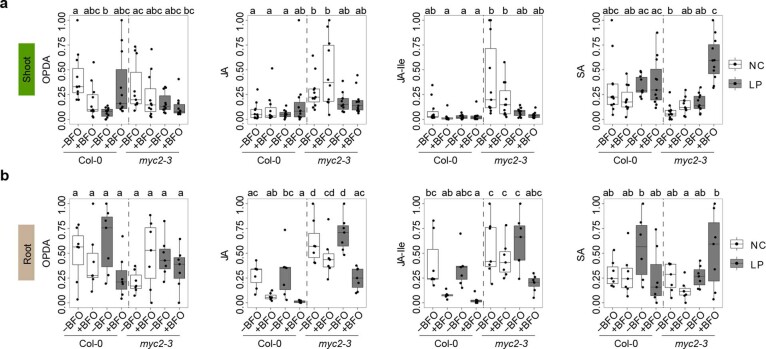
Phytohormone measurements in *A. thaliana* Col-0 and *myc2-3*. Phytohormone levels of shoot (**a**) samples and root (**b**) samples from five-week-old *A. thaliana* Col-0 and *myc2-3* mutants grown under NC and LP, in the absence (-BFO) or presence of the BFO SynCom (+BFO), in the FlowPot system. Phytohormone levels were scaled to 0 – 1 individually. Three independent biological replicates are shown (in shoots: OPDA n = 87 samples, JA n = 87 samples, JA-Ile n = 88 samples, SA n = 87 samples; in roots: OPDA n = 56 samples, JA n = 56 samples, JA-Ile n = 56 samples, SA n = 55 samples). Statistical significance across conditions for each genotype is indicated with letters (Kruskal-Wallis with Dunn’s *post hoc* test, α = 0.05). NC: normal light condition, LP: low PAR. OPDA: 12-oxo-phytodienoic acid, JA: jasmonic acid, JA-Ile: Jasmonic Acid-Isoleucine, SA: free salicylic acid. Boxplot boundaries reflect the inter-quartile range, the centre line is the median, and whiskers represent 1.5× interquartile range of lower and upper quartiles.

Given the fact that MYC2 is a central node controlling the cross-talk between JA and other phytohormone signalling pathways (that is, GA, SA, abscisic acid and indole-3-acetic acid) and regulating responses to light and the circadian clock^49^, we hypothesized that this transcription factor might coordinate the prioritization of microbiota-induced growth over defence under suboptimal light conditions. Importantly, impaired growth and increased resistance to *Bc* observed in the *myc2-3* mutant were quantitatively similar in the independent *myc234* triple mutant (Extended Data Fig. 6o) and reverted in the *jin1-8* pMYC2::MYC2–FLAG line, in which a MYC2–FLAG fusion protein is expressed under control of the native MYC2 promoter (MYC2–FLAG, *myc2* background)^50^ (Fig. 6a and Extended Data Fig. 8a). We also demonstrated that the alleviation of the EODFR-mediated decrease in canopy size by BFO also required MYC2, illustrating that MYC2-dependent rescue of plant growth by BFO is robust across multiple light-limiting conditions, including when light quantity and quality are altered (Fig. 6a). Western blot assays revealed that MYC2 protein abundance in root and shoot samples was modulated by BFO under LP but not under NC (MYC2–FLAG line), with expression levels in the shoot matching canopy size phenotypes (Fig. 6b and Extended Data Fig. 8b). MYC2 protein abundance in roots showed an opposite trend under LP compared with that observed in shoots (Fig. 6b and Extended Data Fig. 8b). We profiled the root and shoot transcriptomes of BFO-colonized and germ-free *myc2-3* and MYC2–FLAG lines exposed to LP and NC in the gnotobiotic FlowPot system five weeks after BFO inoculation. Inspection of 571 shared *MYC2*-regulated genes in shoots and roots under LP validated that MYC2 drives opposite responses between shoots and roots (Extended Data Fig. 8c), which explained the opposite trend of MYC2 protein abundance in shoots and roots (Fig. 6b and Extended Data Fig. 8b). Remarkably, presence/absence of the BFO SynCom explained transcriptome differentiation in shoot samples more than light in the *myc2-3* mutant (Extended Data Fig. 8d), which was validated by PERMANOVA (*myc2-3*, microorganisms: *R*^2^ = 0.394, *P* < 0.001; light: *R*^2^ = 0.179, *P* = 0.006; MYC2–FLAG, microorganisms: *R*^2^ = 0.085, *P* = 0.078; light: *R*^2^ = 0.439, *P* < 0.001; Supplementary Table 6). These results suggest that mutation of *MYC2* resulted either in the attenuation of shoot response to light or in the exacerbation of shoot response to microorganisms. Mutation of *MYC2* led to dramatic shifts in the root and shoot transcriptome, with 5,231 and 5,038 genes differentially regulated in *myc2-3* compared with MYC2–FLAG in roots and shoots, respectively (referred to as *MYC2*-differentially expressed or MDEs, |log_2_FC| ≥ 1, empirical Bayes statistics, FDR < 0.05; Fig. 6c and Extended Data Fig. 8e–g). A large fraction of these MDEs were previously reported as direct targets of MYC2 (refs. ^51–53^) (MDEs root, 52%; MDEs shoot, 58%; Fig. 6d and Extended Data Fig. 9a). Hierarchical clustering of the gene expression profiles of MDEs revealed eight gene expression clusters for both root and shoot samples. In roots, GO term enrichment analyses of MDEs for each cluster revealed that processes related to ion homeostasis (MDER7 and MDER8), circadian rhythm (MDER5), response to sugar (MDER5), defence (MDER1), photosynthesis and response to light (MDER3) or cytokinesis processes (MDER2) were modulated by MYC2 across the different conditions (Extended Data Fig. 8g). In shoots, terms related to photosynthesis (MDES1), defence (MDES2), cytokinesis (MDES3), secondary metabolite biosynthesis (MDES4), response to JA (MDES5) or sugar biosynthesis (MDES7) were also altered in the *myc2-3* mutant (Extended Data Fig. 8g). Notably, clusters MDES2 and MDES7 (shoot) contained GO terms for which the most significant enrichments were observed, and genes in these clusters were either induced (MDES2) or repressed (MDES7) in the *myc2-3* mutant compared with MYC2–FLAG under LP in the presence of BFO (Fig. 6c and Extended Data Fig. 8g). Therefore, MYC2-dependent transcriptional reprogramming between these two clusters probably explained the lack of BFO-mediated growth rescue under LP in the mutant. Inspection of the GO terms and associated genes revealed that BFO-triggered shoot defence responses were retained in the *myc2-3* mutant under LP (MDES2), with 28% of the genes shared between this cluster and the previously identified cluster S1 (Fig. 6e,f; see also Fig. 3b). Consistent with the high levels of free SA (that is, active) measured in leaves of the *myc2*-3 mutant under LP (Extended Data Fig. 10), these responses involved primarily SA-related and/or SAR-related genes (PR1, BGL2, FRK1, EDS5, SARD1, AZI1 and FMO1), indicating that BFO-triggered immunity in leaves remained activated under LP in this mutant (Fig. 6g). In contrast, genes involved in starch and sugar metabolic processes were downregulated under the same condition, illustrating that priority to defence in shoots of the *myc2*-3 mutant was associated with altered sugar metabolic processes. These genes encode enzymes primarily involved in starch biosynthesis (SS1 and SS3), starch accumulation (ADG1 and PGI) or starch breakdown (LSF1, LSF2, PTPKIS1 and BAM3) in shoots (Fig. 6g). Our results indicate that the trade-off between starch/carbon metabolism and defence in shoots is modulated by microbiota and light in a MYC2-dependent manner.

## Discussion

**Fig. 7:**
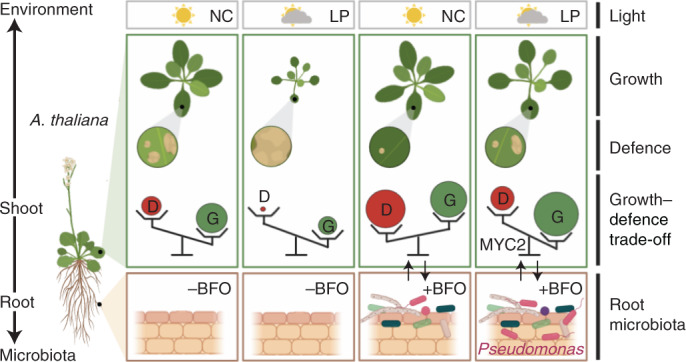
Model for root microbiota-induced growth–defence trade-off between NC and LP. Graphical summary illustrating the bidirectional communication mechanisms along the microbiota–root–shoot–environment axis in *A. thaliana*. In the absence of BFO commensals, plants prioritize growth (G) over defence (D), especially under LP. However, suboptimal light conditions restrict both growth and defence responses, leading to small plants that are highly susceptible to leaf pathogens. In the presence of BFO root commensals under NC, extensive activation of immunity response was observed in leaves, thereby effectively protecting leaves against microbial pathogens. The BFO SynCom also promotes growth responses under this condition, which probably compensates for the fitness cost associated with this elevated immune status. Under suboptimal light conditions, the amplitude of root microbiota-induced systemic immune responses is reduced in a MYC2-dependent manner and growth-promoting bacterial commensals are enriched in roots, thereby boosting plant growth at the expense of defence. Although these plants prioritize microbiota-induced growth over microbiota-induced defence, they still remain more resistant to leaf pathogens than corresponding germ-free control plants grown under NC.

Reminiscent of the bidirectional communication mechanisms described along the microbiota–gut–brain axis in animals^15,54,55^, we report here that aboveground stress responses in plants can be orchestrated through long-distance communication with belowground commensals. We demonstrate that changes in aboveground light can cascade along the shoot–root axis to alter the composition of root commensal communities. Reciprocally, the presence of BFO commensals triggered leaf activation of defence or growth responses in a light-dependent manner. The integration of these responses to microorganisms and light along the root–shoot axis dictates the trade-off between growth and defence, thereby boosting plant growth under suboptimal light conditions (Fig. 7).

Over the course of evolution, the roots of land plants have continuously interacted with multikingdom microbial commensals^2,56^. Consequently, microbial molecules in the rhizosphere (such as hormones, microorganism-associated molecular patterns, volatile organic compounds and lipo-chitooligomers) have been shown to serve as developmental signals for plant growth and immune system maturation^36,57,58^. However, whether microbial signals belowground can modulate aboveground stress responses and vice versa remains poorly understood. Bidirectional communication mechanisms between shoot and root organs have been previously described in the context of aboveground biotic stresses^16^. Leaf colonization by microbial pathogens or herbivores results in shifts in the rhizosphere microbiota through host-induced modulation of root exudation profiles^59–61^. Using manipulation experiments, these pathogen/herbivore-induced shifts in rhizosphere commensal communities were shown to be the direct cause protecting the next plant generation through the promotion of systemic defence responses^11,60,61^. Modulation of the rhizosphere microbiota via leaf-pathogen-induced change in root exudation profiles therefore seems to dictate the survival and performance of the offspring (that is, the cry-for-help hypothesis). Here, we identified a link between differential canopy size across mutants and bacterial community composition under LP and validated that the composition of bacterial root commensals can drive differential investment in microbiota-induced growth or microbiota-induced defence responses. Our results suggest that host-driven recruitment of specific root commensals drives or at least contributes to plant prioritization of growth over defence responses under LP. Although investment in growth under LP is microbiota-dependent, it remains difficult to determine the exact contribution of microbial metabolites, microbiota-induced plant prioritization of growth over defence or microbiota-induced reallocation of resources from root to shoot to the growth-rescue phenotype.

The induction of systemic defence responses, including pathogen‐triggered SAR (SA-dependent) and commensal‐triggered induced systemic resistance (JA- and ethylene-dependent), has been extensively reported in response to specific commensal or pathogenic microorganisms^62–66^. Here we showed that root colonization by a multikingdom consortium of microorganisms originally isolated from the roots of healthy plants triggered aboveground induction of defence responses effective against *Bc* and *Pst*. Compared with germ-free plants, the presence of the BFO SynCom provided extensive protection from leaf pathogens, which was largely independent of the light condition. It is therefore important to note that although a significant part of BFO-induced pathogen resistance is modulated by light and MYC2, most of this protective activity occurred through a MYC2- and light-independent mechanism. Since ectopic leaf colonization by a few root microbiota members was also noted, it is possible that direct antagonism towards pathogens and local defence responses activated in response to these ectopic colonizers also contribute to aboveground pathogen protection. The observed defence responses induced by BFO commensals in the leaves of WT (cluster S1, NC + BFO) and *myc2-3* (cluster MDES2, LP + BFO) plants resemble more stereotypical SAR than induced systemic resistance responses^67^, which is consistent with the fact that induced systemic resistance responses were previously found to be abolished in the *myc2-3* mutant^68^. The induction of immune responses is costly for plants^69,70^, and, consistent with our data (Fig. 6), these responses are known to be associated with the downregulation of genes involved in chloroplast functions, including photosynthetic light reactions, the Calvin cycle, photorespiration and starch metabolism^70–72^. As chloroplasts are central integrators of multiple functions linked to photosynthesis, defence and development^62,63,73^, complex metabolic trade-offs in these organelles probably dictate plant investment into microbiota-induced growth or microbiota-induced defence responses.

A direct link between induced defences and light conditions is supported by previous work showing an absolute requirement of phytochromes, but not cryptochromes, for biological induction of SAR^22,74^. Since phytochrome-mediated light signalling is interconnected with JA signalling, probably via MYC2 (refs. ^24,75–77^), it has been suggested that MYC2 might orchestrate the regulation of plant growth and development by light quality^49^. Our results are consistent with this hypothesis and suggest that LP-dependent downregulation of microbiota-induced defence in leaves is orchestrated through MYC2 via a cross-talk between photoreceptor signalling and defence signalling. Our observation that MYC2 is needed for LP-triggered reduction of immune activity against *Pst* and *Bc* in the presence of BFO commensals but not under axenic conditions indicates that this MYC2-dependent growth–defence trade-off requires signals from microbial commensals. This result further supports the hypothesis that, over evolutionary time, the direct integration of microbial and environmental cues by plants has been key for plant adaptation to environmental constraints.

Our results suggest that the interference of MYC2 (the JA signalling pathway) with light, SA/SAR and GA signalling pathways is key to prioritizing investment in shoot growth over shoot defence under LP. In our gene expression network (Fig. 6), we identified a few genes connecting the clusters MDES2 (defence cluster) and MDES7 (growth cluster). These MDEs include EDS5 (ref. ^78^), a transporter required for SA export from chloroplasts and needed for SA signalling (induced in *myc2-3* in LP); AOS^79^, a key chloroplastic enzyme needed for JA biosynthesis (repressed in *myc2-3* in LP); and RGL3 (ref. ^80^), a repressor of GA responses (induced in *myc2-3* in LP). As these genes are well-known MYC2 target genes^51,53,81,82^, it is conceivable that differential regulation of the expression of these genes by MYC2 orchestrates a complex cross-talk between JA, SA and GA to prioritize either microbiota-induced growth or defence according to the surrounding light conditions. Importantly, this survival trade-off might not necessarily occur in plant species that naturally grow at high density or under suboptimal light conditions, as exemplified by the concomitant activation of defence and shade avoidance responses observed in *Geranium robertianum*^83^.

Taken together, our data suggest that plant growth and survival in nature probably depend on the ability of these sessile organisms to utilize belowground microbial signals to prioritize either growth or defence depending on light quantity and quality perceived by leaves. Phenotypic plasticity and aboveground stress responses in plants can therefore be governed by microbial root commensals.

## Methods

All experiments were performed at least three independent times, except RNA-seq experiments, which were carried out once with three biological replicates. All findings were consistent across replicates.

### Microbial strains and culture conditions

The bacterial, fungal and oomycete strains used in this study have been previously reported^12,35^ and are summarized in Supplementary Table 1. *Pst* and *Bc* (a benomyl derivative of the strain SAS56) were used as model pathogens for pathogen assays in *A. thaliana*^48,84^. Bacterial strains were routinely cultured at 25 °C in liquid 50% Tryptic Soy Broth (Sigma-Aldrich) and stored at −80 °C in 25% glycerol. Fungal strains were cultured at 20 °C in solid PGA media (Sigma-Aldrich), and agar plugs containing mycelia were stored at −80 °C in 30% glycerol. Oomycete strains were continuously propagated in solid PGA media. *Pst* was cultured at 28 °C in NYGA medium and stored at −80 °C in 7% dimethyl sulfoxide. *Bc* spores were obtained from −80 °C glycerol stocks in which the concentration was adjusted to 10^7^ spores per ml in Vogelbuffer (in 1 l: 15 g sucrose, 3 g Na-citrate, 5 g K_2_HPO_4_, 0.2 g MgSO_4_7H_2_O, 0.1 g CaCl_2_2H_2_O, 2 g NH_4_NO_3_).

### Plant material and growth conditions

*A. thaliana* Col-0 and the mutants used in this study are provided in this section and Supplementary Table 4. *A. thaliana* Col-0 WT (N60000) was obtained from the Nottingham Arabidopsis Stock Centre. The *dellaP* mutant (*della* pentuple) was previously generated by crossing *ga-28*, *rgl1-SK62*, *rgl2-SK54*, *rgl3-3* and introgressed *gai-t6* (ref. ^40^). The *jazQ* mutant (*jaz* quintuple) was previously obtained from T-DNA insertion mutants of *jaz1-2*, *jaz3-4*, *jaz4-1*, *jaz9-4* and *jaz10-1* (ref. ^24^). The mutants *dde2-2* (CS65993)^38^, s*id2-2* (CS16438)^37^, *myc2-3* (salk_061267)^39^, *sav3-3* (ref. ^43^), *bri1-301* (ref. ^41^), *myc234* (ref. ^85^), *cry1-304 cry2-1* (ref. ^86^) and *jin1-8* pMYC2::MYC2–FLAG^50^ were previously reported. Seeds of *A. thaliana* Col-0 and mutant plants were surface-sterilized in 70% ethanol for 18 min followed by a brief wash with 100% ethanol (1 min). The seeds were dried out under sterile bench conditions and were incubated for two days at 4 °C in the dark. Individual seeds were sown onto the surface of FlowPots^30^ by pipetting one seed at a time. Before seed sowing, the FlowPots were inoculated with half-strength Murashige and Skoog medium (without sucrose, pH 5.5; Sigma-Aldrich) with or without microbial commensals. Combined boxes containing FlowPots with sterile or colonized plants were incubated under short-day conditions at 21 °C with three light conditions (NC: photosynthetic photon flux density, 62.35 µmol m^−2^ s^−1^; LP: photosynthetic photon flux density, 27.91 µmol m^−^^2^ s^−1^; EODFR: 15 min far-red light (740 nm) treatment at the end of the day) (10 h) and at 19 °C in the dark (14 h). The light condition was measured by Spectral PAR meter PG100N (UPRtek).

### Microbiota reconstitution experiments in the FlowPot system

The bacterial strains were cultivated in 96-deep-well plates containing 400 μl of 50% Tryptic Soy Broth (Sigma-Aldrich) with 180 r.p.m. shaking speed for seven days at 25 °C and subsequently pooled at equal volume ratios. This bacterial pool was centrifuged at 4,000 *g* for 10 min and resuspended in 10 mM MgCl_2_ to remove residual media and bacteria-derived metabolites. Prior to inoculation, OD_600_ was adjusted to 0.5. Individual fungal and oomycete strains were cultivated on solid PGA medium for 14 days. Then, 100 mg of fungal and 40 mg of oomycete mycelium was harvested for each strain and aliquoted into 2 ml Eppendorf tubes containing 1 ml of MgCl_2_ and one sterile stainless-steel bead (3.2 mm). The mycelium was subsequently crushed with a paint shaker (SK450, Fast & Fluid Management) for 10 min. Fragmented fungal or oomycete mycelia were then pooled at equal volume ratios at a concentration of 100 mg ml^−1^ for fungi and 40 mg ml^−1^ for oomycetes. For the microbial ratio experiment, either 2 ml (high concentration) or 0.2 ml (low concentration) of the bacterial suspension (OD_600_ = 0.5; see above), 1 ml (high) or 0.1 ml (low) of the fungal suspension (100 mg ml^−1^; see above), and 1 ml (high) or 0.1 ml (low) of the oomycete suspension (40 mg ml^−1^; see above) was transferred into a falcon containing 50 ml of Murashige and Skoog medium without sucrose, which was used to repopulate sterile peat in the FlowPot. For all the other experiments in the FlowPot system, 0.2 ml (low) of the bacterial suspension (OD_600_ = 0.5), 0.1 ml (low) of the fungal suspension (100 mg ml^−1^) and 0.1 ml (low) of the oomycete suspension (40 mg ml^−1^) were used. The procedures for setting up the gnotobiotic FlowPot system were carried out as previously described^12,30^. One week after incubation, the seedlings were randomly thinned out under a sterile bench to keep three plants per FlowPot. The plants were harvested at the vegetative stage five weeks after seed sowing for all experiments.

### CAS soil and CAS wash repopulation experiments in the FlowPot system

The natural CAS mixed with sterile vermiculites (volume of soil:vermiculite, 2:1) was used as the matrix in the FlowPot. The procedures for setting up the FlowPot system were carried out as described above. The properties of CAS soil and peat were measured by Labor für Boden- und Umweltanalytik and are provided in Supplementary Table 1. For the CAS wash, 10 ml of soil was washed with a sterile detergent (sterile 1× TE + 0.1% Triton X-100) in a 1:10 ratio (soil:detergent) in a 50 ml falcon tube and shaken vigorously until the soil pellet was well mixed. The tube was then placed in a rotator for 30 min at 40 r.p.m. at room temperature. To remove big soil particles, the tube was centrifuged for 1 min at 1,500 r.p.m. The supernatant was transferred to a new falcon tube and centrifuged for 20 min at 4,000 r.p.m. The supernatant was then discarded, and the pellet was resuspended in the same initial volume of 10 mM MgCl_2_ as the CAS wash. Then the CAS wash was repopulated in the sterile peat matrix. Meanwhile, a heat-killed soil wash (CAS wash HK) was prepared by incubating it at 95 °C for 45 min. The sterility of the heat-killed soil wash was validated by plating it on TSA and PGA plates. The other procedures for setting up the FlowPot system were carried out as described above.

### Shoot trait measurements

Shoots of individual plants were cut, and their fresh weight was measured first. The shoots were placed on white paper, sealed with polyester non-sterile transparent film (VWR) and scanned (Perfection V600 Photo, Epson). Canopy size, petiole length, leaf length, leaf width, leaf numbers and hypocotyl length were measured using ImageJ (Fiji)^87^.

### Microbial community profiling

For community profiling, plant roots (a pool of three root systems per FlowPot) and shoots were thoroughly washed with sterile Milli-Q water and dried out with sterilized Whatman glass microfibre filters (GE Healthcare Life Sciences). Matrix samples, corresponding to peat substrate in the pot without plant roots, were also harvested (around 0.5 ml of peat per FlowPot). Plant roots, shoots and matrix samples were then transferred into individual lysing matrix E tubes (MP Biomedicals), frozen in liquid nitrogen and stored at −80 °C. The samples were crushed using the Precellys 24 tissue lyser at 6,200 r.p.m. for 30 s (Bertin Technologies), and DNA isolation was performed using the FastDNA SPIN for soil kit (MP Biomedicals), as previously described^12^. DNA concentration was quantified using the Quant-iT PicoGreen dsDNA assay kit (Invitrogen), and the fluorescence of dsDNA was measured by qPCR (IQ5 real-time PCR Thermocycler, Biorad) using the following parameters: 30 s at 25 °C, 3 × 30 s at 25 °C for measuring fluorescence, 30 s at 15 °C. Sample concentration was adjusted to 3.5 ng μl^−1^ with sterile water. Library preparation of individual samples involved a two-step PCR protocol. In the first PCR step (20 cycles)^12^, the 25 μl of reaction mix contained 3 μl of sample DNA, 1× incomplete reaction buffer, 1.25 U DFS-Taq DNA Polymerase, 2 mM MgCl_2_, 0.3% BSA, 200 μM dNTPs and 400 nM of each primer. Universal primers targeting the bacterial 16S rRNA V5-V6 region (799F/1192R), the fungal ITS1 (ITS1F/ITS2) and the oomycete ITS1 (ITS1-O/5.8s-O-Rev) were used, as previously described^12^. After the first PCR, the enzymes and ssDNA were digested using a mixture of 1 μl of Antarctic phosphatase, 1 μl of Exonuclease I and 2.44 μl of Antarctic phosphatase buffer that was added to 20 μl of the first-step PCR product (37 °C for 30 min and 85 °C for 15 min). After centrifugation, 3 μl of this solution was used as a template for a second PCR (ten cycles)^12^. This step involved the same aforementioned PCR mix except that the universal primers were barcoded (reverse primers only) and included P5 and P7 Illumina adaptors. After PCR, 5 µl of PCR product for each barcoded sample was mixed with 5 µl of 6× Orange G DNA Loading Dye (Orange G, 6×, Sigma) and run on a 1% agarose gel in TAE 1× buffer. After control gel checking, the remaining PCR product (around 70 μl) was mixed with 20 μl of Gel Loading Dye (Orange G) and run on a 1.5% agarose gel for 2 h at 80 V to excise the bacterial 16S rRNA band (500 base pairs (bp)). The bands were cut and purified using the QIAquick gel extraction kit (QIAGEN), and DNA concentration was measured using the PicoGreen method as described above. For fungi and oomycetes, DNA purification was performed using the Agencourt AMPure XP-PCR Purification method (Beckman Coulter). DNA sample concentration was then quantified by the Quant-iT PicoGreen dsDNA method, as described above. For each microbial group (B, F and O), 30 ng of DNA of each of the barcoded samples were mixed, resulting in three pooled samples that were purified twice (AMPure XP-PCR Purification, Beckman Coulter). The final DNA concentration of each pool was measured using the Quantus Fluorometer (Promega), and an equal quantity of DNA (that is, 100 ng) was used to assemble a single library from the three bacterial, fungal and oomycete DNA pools. Finally, paired-end Illumina DNA sequencing was performed using the Illumina MiSeq system available on-site at the Max Planck Institute for Plant Breeding Research.

### 16S rRNA and ITS read processing

Paired amplicon sequencing reads were joined using Qiime (v.1.9.1) (join_paired_reads)^88^. In the case of ITS reads, for unjoined read pairs, corresponding forward reads were retained for demultiplexing. Demultiplexing and quality filtering were done using Qiime (split_libraries_fastq, phred = 30). All quality filtered and demultiplexed reads were trimmed to an equal length. Reference sequences were obtained from all used strains (183 bacteria, 24 fungi and 7 oomycetes; see Supplementary Table 1) using the respective resources of sequenced genomes, if available. The reference sequences were then trimmed to contain only the amplified region. Trimmed reference sequences that were 100% identical were grouped in so-called strain variants. This led to 115 bacterial, 24 fungal and 7 oomycete strain variant sequences. The reference sequences of strain variants were mapped against all trimmed amplicon reads using usearch (v.8.0.1517)^89^, allowing for one mismatch (usearch –usearch_global, with max diff = 1). Unmapped reads were discarded. Count tables were made from these mapping results.

### Microbial community profile statistical analysis

To calculate alpha diversity indices, the count tables were rarefied to 1,000 reads. For the microbial profiling data, only samples with more than 1,000 reads were considered. Significant differences between alpha diversity indices were determined by an ANOVA followed by post hoc Tukey’s HSD (*P* < 0.05). Distance matrices were calculated by using a normalized count table (CSS)^90^, which was used for Bray–Curtis distance calculation. Distance tables were used as an input for PCoA and CAP (capscale function in R). The condition function was used to correct for batch effects and when applicable for treatments (LP or NC) when performing CAP. The significance of the CAP results was tested using ANOVA (anova.cca in R, *P* < 0.05). PLS-DA was used to discriminate groups of samples on the basis of the composition of their microbial communities. PLS-DA consists of a partial least squares regression analysis where the response variable is categorical (*y*-block; describing the grouping factor), expressing the class membership of the statistical units^91^. Raw operational taxonomic unit tables were first scaled before using the cppls function in the package pls. The PLS-DA procedure includes a cross-validation step producing a *P* value that expresses the validity of the PLS-DA method regarding the dataset (function MVA.test in package RVAideMemoire). The PLS-DA procedure also expresses the statistical sensitivity, indicating the modelling efficiency in the form of the percentage of misclassification of samples in categories accepted by the class model (function MVA.cmv in package RVAideMemoire). To better plot differences in abundances of individual strains across different sets of experiments, RAs were rescaled to be in a range from 0 to 1. For this, read counts per sample were transformed to RAs by division of the total sum of reads per sample. Then, for all samples belonging to one experiment, the RA of each individual strain variant across the desired samples (for example, only root samples or only WT and mutant samples) was rescaled using the general equation *x* − [min(*x*)]/[max(*x*) − min(*x*)], where *x* represents all RA values per strain variant from one experiment. Strain variants that were not consistently found across samples were discarded from this analysis.

The enrichment of strain variants across LP and NC conditions was calculated using the following steps (all functions are from the R package EdgeR). Raw read counts were normalized using TMM normalization (calcNormFactors). A generalized linear model was fitted to integrate the batch effect (glmFit). Enrichment was then determined with a likelihood ratio test (glmLRT, *P* < 0.05). A predictive SVM model with a linear kernel was trained (SVC function of Scikit-learn)^92^ to link standardized RAs of bacteria to plant rescue. Recursive feature elimination with cross-validation (RFECV function) was performed to identify the smallest set of bacteria discriminating the two plant phenotypes and to estimate the model accuracy using a leave-one-out approach (*K*-fold cross-validator, with *K* equalling the number of samples). The species identified in the RFECV were then used to compute a PLS-DA.

### Transcriptome sequencing experiments

Shoots and roots of five-week-old *A. thaliana* Col-0, *myc2-3* and *jin1-8* pMYC2::MYC2–FLAG plants growing in the FlowPot system were harvested separately at 10 a.m. The roots were washed quickly (<1 min) to detach the surrounding peat matrix using 10% RNAlater (QIAGEN) in 1× PBS as the capture buffer to mitigate RNA degradation. Total RNA from all samples was extracted using the RNeasy Plant Mini Kit (QIAGEN). DNA removal was performed using RNase-Free DNase Set (QIAGEN). Eleven samples from *myc2-3* plants detected more than 50% reads mapped to latent genome. On the basis of the hierarchical relationship between samples from *myc2-3* mutants (Extended Data Fig. 8b), the contamination with latent virus did not significantly affect the plant transcriptome. RNA-seq libraries were prepared and sequenced at the Max Planck Genome Centre with an Illumina HiSeq2500. For samples from Col-0 plants, the run conditions were 1 × 150 bp (single read), and the total reads per sample were 20 million. For samples from *myc2-3* plants, the run conditions were 1 × 150 bp (single read), and the total reads per sample were 55 million. For samples from *jin1-8* pMYC2::MYC2–FLAG plants, the run conditions were 1 × 150 bp (single read), and the total reads per sample were 10 million.

### Transcriptome sequencing data analysis

The FastQC suite (http://www.bioinformatics.babraham.ac.uk/projects/fastqc/) was performed to check the quality of the sequenced reads. The RNA-seq reads were then mapped to the annotated genome of *A. thaliana* (TAIR10) using Tophat2 (v.2.1.1) (tophat2 parameters: "p, 20; a, 10; g, 10')^93^ with Bowtie2 (ref. ^94^) (v.2.2.3) building the genome index. The mapped RNA-seq reads were subsequently transformed into a fragment count per gene per sample using the htseq-count script (s, reverse; t, exon) in the package HTSeq^95^ (v.0.11.2). Count tables of Col-0 samples were concatenated to one count matrix. Count tables of *myc2-3* and *jin1-8* pMYC2::MYC2–FLAG samples were concatenated to one count matrix. Raw counts were normalized via TMM normalization. Then, the genes with more than 100 counts were extracted. The extracted TMM-normalized count data were transformed to log_2_cpm via the voom function in the limma package^96^ in R (R v.3.6.3; https://www.r-project.org/). Subsequently, the log_2_cpm data were used to calculate log_2_ fold changes and *P* values (*F*-test) for individual comparisons. The resulting *P* values were adjusted for false discoveries due to multiple hypothesis testing via the Benjamini–Hochberg procedure. To identify significantly differently expressed genes, a threshold of |log_2_FC| ≥ 1 and FDR < 0.05 was applied. Heat maps of the expression profiles of significantly regulated genes were generated using the pheatmap package in R. The Euclidean distance was used to show the distance among clustering rows. The values in the heat maps were scaled by row. GO enrichment was conducted using the enrichGO function in the clusterProfiler package^97^ in R. Biological process and 0.05 *P* value cut-offs were chosen. The *P* values were adjusted via the Benjamini–Hochberg method.

### Variance partitioning of microbial community composition and transcriptomic profiles

Variance partitioning between experimental factors was tested with a permutational ANOVA approach with the Adonis function (R package vegan) in all experiments. For the stress experiment, the effect of compartment and light condition factors on microbial community composition was tested in a global model. The effect of light conditions was also tested in separate models for root and shoot samples. These models were constructed separately on each of the bacterial, fungal and oomycete datasets using Bray–Curtis dissimilarity matrices between pairs of samples produced with the vegdist function (R package vegan). For the mutant experiment, the effects of genotype, compartment and light condition factors on bacterial community composition were tested in a global model. The effects of genotype and light conditions were also tested in separate models of root and matrix samples. These models were constructed using Bray–Curtis dissimilarity matrices between pairs of samples produced with the vegdist function (R package vegan). For the transcriptomic experiments, the effects of the experimental factors on the transcriptomic expression profiles of *A. thaliana* plants were tested using Euclidean distance matrices between pairs of samples produced with the vegdist function (R package vegan). Models were constructed separately for Col-0 plants and for *myc2-3* and *jin1-8* pMYC2::MYC2–FLAG plants, as these were harvested in separate experiments. For Col-0 plants, the effects of compartment, microorganism inoculation and light conditions on the plant transcriptomic profile were tested in a global model. The effects of microorganism inoculation and light conditions were also tested in separate models for root and shoot samples. For *myc2-3* and *jin1-8* pMYC2::MYC2–FLAG plants, the effects of compartment, genotype, microorganism inoculation and light conditions on the plant transcriptomic profile were tested in a global model. The effects of genotype, microorganisms and light were also tested in separate models for root and shoot samples. These models were also constructed for the *myc2-3* and *jin1-8* pMYC2::MYC2–FLAG datasets separately to test the effects of compartment, microorganism inoculation and light independently of the genotype effect.

### Pathogen inoculation and symptom quantification

For *Bc* inoculation of *A. thaliana*, the spores were diluted in Vogelbuffer (in 1 l: 15 g of Suc, 3 g of Na-citrate, 5 g of K_2_HPO_4_, 0.2 g of MgSO_4_·7H_2_O, 0.1 g of CaCl_2_·2H_2_O and 2 g of NH_4_NO_3_) to 5 × 10^5^ spores per ml. For droplet inoculations, 2 μl droplets containing 1 × 10^3^ spores were applied to each leaf of four-week-old *A. thaliana* grown in the presence or absence of the BFO SynCom under either NC or LP. The entire infection process was conducted under a sterile clean bench. Five days after pathogen inoculation in the FlowPot system, the shoots were cut, washed using Milli-Q water twice and dried out with sterilized Whatman glass microfibre filters (GE Healthcare Life Sciences). The shoots were weighed, placed into 2 ml sterile Eppendorf tubes and snap-frozen in liquid nitrogen. For quantification of fungal growth, DNA from the plant shoots was extracted using the DNeasy Plant Mini Kit (QIAGEN). The relative amounts of *Bc* and *A. thalian*a DNA were determined by qPCR, employing specific primers for cutinase A and SKII, respectively, as described previously^48^. *Pst* inoculation was carried out by spraying *Pst* at 0.2 OD in 10 mM MgCl_2_ on the leaves of four-week-old *A. thaliana* grown in the presence or absence of the BFO SynCom under either NC or LP. The entire infection process was conducted under a sterile clean bench. Five days after pathogen inoculation in the FlowPot system, the plant shoots were cut, washed using 70% ethanol once and rinsed in Milli-Q water twice. The shoots were dried with sterilized Whatman glass microfibre filters and weighed under a sterile clean bench. Then, the shoots were put into 2 ml sterile Eppendorf tubes containing 1.5 ml of 10 mM MgCl_2_/0.01% Silwet. The tubes were then directly shaken at 650 r.p.m. for 1 h at 28 °C. For colony counting, a series of dilutions were conducted with 10 mM MgCl_2_ to 10^−1^,10^−2^,10^−3^,10^−4^ and 10^−5^. Then, 20 μl of diluted liquid was spotted on NYGA plates, and colonies were counted after two days of incubation at 28 °C.

### Immunoblot analysis

To analyse MYC2 protein levels of plants under NC or LP in the presence or absence of BFO, the shoots and roots of *A. thaliana* Col-0 and *jin1-8* pMYC2::MYC2–FLAG plants (grown in the FlowPot system under NC or LP conditions as described above) were harvested separately into 2 ml Eppendorf tubes with one 3.2 mm stainless-steel bead five weeks after −BFO or +BFO inoculation and frozen in liquid nitrogen. Samples with the same fresh weight were ground to powder in liquid nitrogen and boiled in 150 μl of 2× Laemmli buffer for 10 min at 95 °C. The proteins were resolved on 10% SDS–PAGE (1610156, Bio-Rad) and transferred using the wet transfer method onto a nitrocellulose membrane (10600001, GE Healthcare Life Sciences). Monoclonal anti-β-actin-peroxidase antibody produced in mouse (A3854, Sigma-Aldrich; clone: AC-15, monoclonal) was used to detect actin and to adjust the total protein concentration. Monoclonal anti-FLAG M2-peroxidase antibody produced in mouse (A8592, Sigma-Aldrich; clone: M2, monoclonal) was used to detect MYC2 protein. Both antibodies were used at a dilution of 1:5,000 (1× TBST, 5% milk powder). First, a full membrane was used to check MYC2 and actin separately. Then, the membranes performed later were cut at 50 kDa to separate MYC2 (roughly 75 kDa) and actin (roughly 40 kDa) to detect both proteins together. Detection of the signal was performed with SuperSignal West Pico and Femto (34080 and 34095, ThermoFisher Scientific) using ChemiDoc Imaging Systems (Bio-Rad). The protein concentration was calculated on the basis of the band thickness using Fiji.

### Phytohormone measurement

To measure phytohormones of plants under NC or LP in the presence or absence of BFO, the shoots and roots of *A. thaliana* Col-0 and *myc2-3* plants (grown in the FlowPot system under NC or LP conditions as described above) were harvested separately into 2 ml Eppendorf tubes with one 3.2 mm stainless-steel bead. Phytohormone concentrations were determined from 50 mg of tissue (fresh weight). Sample processing, data acquisition, instrumental set-up and quantifications (using 5 ng of [^2^H]_6_ JA, 0.75 ng of [^2^H]_2_-JA-Ile, 30 ng of [^2^H]_5_-OPDA and 1.5 ng of [^2^H]_4_-SA as internal standards per sample) were performed as described in refs. ^98,99^.

### Quantification and statistical analysis

Data collection and analysis were performed under blinded conditions in all experiments. All statistical analyses were performed using the R environment (R v.3.6.3).

### Reporting Summary

Further information on research design is available in the Nature Research Reporting Summary linked to this article.

## Supplementary information

Reporting Summary

Supplementary Tables Supplementary Table 1: Microbial culture collections used for microbiota reconstitution experiments and properties of CAS soil and peat. Supplementary Table 2: PERMANOVA partitioning of microbial community assemblages. Supplementary Table 3: RNA-seq expression profiling of *A. thaliana* genes in root and shoot samples. Supplementary Table 4: *A. thaliana* mutants used in this study. Supplementary Table 5: PERMANOVA partitioning of microbial community assemblages across *A. thaliana* mutants and the bacteria strain variants identified by the SVM. Supplementary Table 6: RNA-seq expression profiling of *A. thaliana* genes (*myc2-3* and *jin1-8* pMYC2::MYC2–FLAG) in root and shoot samples.

## Data Availability

The sequencing reads from the microbiota reconstitution experiments (MiSeq 16S rRNA and ITS reads) have been deposited in the European Nucleotide Archive (bacteria at PRJEB40980, fungi at PRJEB40981 and oomycetes at PRJEB40982). The sequencing reads from the transcriptome sequencing experiments have been deposited in the Gene Expression Omnibus (Col-0 data at GSE160106 and *myc2-3* and *jin1-8* pMYC2::MYC2–FLAG data at GSE160115). All raw data in this study are available at https://github.com/ShijiHou/Light-limitation-Paper.

## Code availability

All scripts for computational analysis are available at https://github.com/ShijiHou/Light-limitation-Paper.
