# Supplementary material for: A microbiota–root–shoot circuit favours Arabidopsis growth over defence under suboptimal light
Source: Nat Plants. 2021 Jul 5;7(8):1078–92. doi: 10.1038/s41477-021-00956-4 (PMC8367822; doi:10.1038/s41477-021-00956-4)
Supplement: Supplementary file 1 — Reporting Summary [file 41477_2021_956_MOESM1_ESM.pdf]

nature research

Corresponding author(s): Dr. Stéphane Hacquard

Last updated by author(s): May 27, 2021

Reporting Summary

Nature Research wishes to improve the reproducibility of the work that we publish. This form provides structure for consistency and transparency in reporting. For further information on Nature Research policies, see our Editorial Policies and the Editorial Policy Checklist.

Statistics

For all statistical analyses, confirm that the following items are present in the figure legend, table legend, main text, or Methods section.

n/a	Confirmed	
☐	☒	The exact sample size (*n*) for each experimental group/condition, given as a discrete number and unit of measurement
☐	☒	A statement on whether measurements were taken from distinct samples or whether the same sample was measured repeatedly
☐	☒	The statistical test(s) used AND whether they are one- or two-sided<br>*Only common tests should be described solely by name; describe more complex techniques in the Methods section.*
☐	☒	A description of all covariates tested
☐	☒	A description of any assumptions or corrections, such as tests of normality and adjustment for multiple comparisons
☐	☒	A full description of the statistical parameters including central tendency (e.g. means) or other basic estimates (e.g. regression coefficient) AND variation (e.g. standard deviation) or associated estimates of uncertainty (e.g. confidence intervals)
☐	☒	For null hypothesis testing, the test statistic (e.g. *F*, *t*, *r*) with confidence intervals, effect sizes, degrees of freedom and *P* value noted<br>*Give P values as exact values whenever suitable.*
☒	☐	For Bayesian analysis, information on the choice of priors and Markov chain Monte Carlo settings
☐	☒	For hierarchical and complex designs, identification of the appropriate level for tests and full reporting of outcomes
☐	☒	Estimates of effect sizes (e.g. Cohen's *d*, Pearson's *r*), indicating how they were calculated

Our web collection on statistics for biologists contains articles on many of the points above.

Software and code

Policy information about availability of computer code

Data collection	ImageJ (Fiji) was used to measure canopy size, Petiole length, leaf length, leaf width and quantify the band thickness of western blot.
Data analysis	R (version 3.6.3) was used for data transformation, analysis, and visualization. Qiime (v1.9.1, http://qiime.org/) and Usearch (v8.0.1517, https://www.drive5.com/usearch/) were used to process 16S rRNA and ITS read alignment. DADA2 (version 1.12.1) and Vegan (version 2.5.6) were used for denoising and analyzing 16s rRNA amplicon reads, and community profiling analysis diversity analyses. edgeR (version 3.24.3) was used to identify differentially enriched Sequence Variants. tidyverse (version 1.2.1) was used for data formating. RNASeq reads were checked with fastqc suite (http://www.bioinformatics.babraham.ac.uk/projects/fastqc/), aligned using Tophat2 (version 2.1.1) and Bowtie2 (version 2.2.3), and normalized by limma package (version 3.42.2). pheatmap package (version 1.0.12) was used to generate heatmaps. Gene ontology analysis was performed with the clusterProfiler package (version 3.14.3).<br>ggplot2 (version 3.3.0) and ggpubr (version 0.4.0) were used for data visualization. Final figures were prepared in Adobe Illustrator 2020 for combining, positioning, and resizing plot and text.<br>Code & raw data: https://github.com/ShijiHou/Light-limitation-Paper

For manuscripts utilizing custom algorithms or software that are central to the research but not yet described in published literature, software must be made available to editors and reviewers. We strongly encourage code deposition in a community repository (e.g. GitHub). See the Nature Research guidelines for submitting code & software for further information.

Data

Policy information about availability of data

All manuscripts must include a data availability statement. This statement should provide the following information, where applicable:

- Accession codes, unique identifiers, or web links for publicly available datasets
- A list of figures that have associated raw data
- A description of any restrictions on data availability

Sequencing reads from microbiota reconstitution experiments (MiSeq 16S rRNA and ITS reads) have been deposited in European Nucleotide Archive (ENA, PRJEB40980 – bacteria at https://www.ebi.ac.uk/ena/browser/view/PRJEB40980, PRJEB40981 – fungi at https://www.ebi.ac.uk/ena/browser/view/PRJEB40981, PRJEB40982 – oomycetes at https://www.ebi.ac.uk/ena/browser/view/PRJEB40982). Sequencing reads from transcriptome sequencing experiments have been deposited in Gene Expression Omnibus (GEO, GSE160106 – Col-0 data at https://www.ncbi.nlm.nih.gov/geo/query/acc.cgi?acc=GSE160106, GSE160115 – myc2-3 and jin1-8 pMYC2:MYC2-FLAG data at https://www.ncbi.nlm.nih.gov/geo/query/acc.cgi?acc=GSE160115). All raw data in this study are available at https://github.com/ShijiHou/Light-limitation-Paper.

Field-specific reporting

Please select the one below that is the best fit for your research. If you are not sure, read the appropriate sections before making your selection.

☒ Life sciences ☐ Behavioural & social sciences ☐ Ecological, evolutionary & environmental sciences

For a reference copy of the document with all sections, see nature.com/documents/nr-reporting-summary-flat.pdf

Life sciences study design

All studies must disclose on these points even when the disclosure is negative.

Sample size	Statistical methods were not used to predetermine necessary sample size. Rather, efforts were made to ensure large replicate numbers per experiment (usually n>24 per experiment), and replicate experiments were performed.<br>For microbial profiling analyses, root samples from six plants grown in two single pots were pooled to create a single sample. Soil samples from two single pot were pooled to create a single sample. We aimed to have at least three root samples and three soil samples per treatment, and three independent biological replicate experiments. Overall, for each genotype and each condition, 18 samples (9 samples from roots and 9 samples from matrixes) were chosen from 3 independent biological replicates (3 technical replicates per independent biological replicate).<br>For RNASeq experiments, samples (shoot and root) from 12 plants grown in four single pots were pooled to create a single sample. In total, we had three root samples and three shoot samples per treatment.<br>For microbiota reconstitution and plant growth assays experiments, experiments were performed with n=54 plants per condition, split across 3 replicate plates each containing 18 individual plants. In this way, each experiment contained replicate microbiota reconstitution conditions (n=3 plates) and n=18 plants per plate. Experiments were repeated at least three times and the results pooled.
Data exclusions	Very few samples where 16S rRNA or ITS sequences did not meet predetermined quality checks (i.e. low quality or low read counts) were excluded. Very few samples where the leaf was broken during harvesting were excluded for measuring leaf traits.
Replication	Experiments were performed at least three independent times to confirm reproducibility. All findings from replicate experiments were consistent and confirmed.
Randomization	Seeds of each genotype were allocated into different conditions at random without note of the different conditions. Each replicate pot or plate of each condition of the experiment was distributed at random into the growth chamber, and randomly shuffled every two to three days to minimize location-based effects. Samples were collected randomly for each condition in all experiments.
Blinding	During sample harvesting and measuring shoot fresh weight, no procedure was taken to blind experimenters to the different conditions since different light treatments are obviously distinguished. Harvested tissues were stored in numbered tubes without sample condition or identity labels. The sample metadata was stored in a separate file. Thereby, during the processing of samples for microbial profiling, RNASeq analysis, leaf traits, leaf pathogen quantification, and phytohormone content, experimenters did not know the identity of the samples.

Reporting for specific materials, systems and methods

We require information from authors about some types of materials, experimental systems and methods used in many studies. Here, indicate whether each material, system or method listed is relevant to your study. If you are not sure if a list item applies to your research, read the appropriate section before selecting a response.

Materials & experimental systems

n/a	Involved in the study
☐	☒ Antibodies
☒	☐ Eukaryotic cell lines
☒	☐ Palaeontology and archaeology
☒	☐ Animals and other organisms
☒	☐ Human research participants
☒	☐ Clinical data
☒	☐ Dual use research of concern

Methods

n/a	Involved in the study
☒	☐ ChIP-seq
☒	☐ Flow cytometry
☒	☐ MRI-based neuroimaging

Antibodies

Antibodies used

Monoclonal Anti-β-Actin-Peroxidase antibody produced in mouse (A3854, Sigma-Aldrich, Germany; Clone: AC-15, monoclonal).
Monoclonal ANTI-FLAG® M2-Peroxidase antibody produced in mouse (A8592, Sigma-Aldrich, Germany; Clone: M2, monoclonal).

Validation

Monoclonal Anti-β-Actin-Peroxidase antibody produced in mouse (A3854, Sigma-Aldrich, Germany; Clone: AC-15, monoclonal) was used to detect Actin in Arabidopsis thaliana. This primary antibody is peroxidase conjugated. The validation information is available at https://www.sigmaaldrich.com/catalog/product/sigma/a3854?lang=de&region=DE&gclid=CjwKCAjwtJ2FBhAuEiwAIKu19hRcDr2tZZAQ9NvvxZiOyR0KjOJr9GCViO3eVO5rvvmI1l0yr07ZqRoCvTYQAvD_BwE.
Monoclonal ANTI-FLAG® M2-Peroxidase antibody produced in mouse (A8592, Sigma-Aldrich, Germany; Clone: M2, monoclonal) was used to detect MYC2 protein in Arabidopsis thaliana jin1-8 pMYC2:MYC2-FLAG line. This primary antibody is peroxidase conjugated. The validation information is available at https://www.sigmaaldrich.com/catalog/product/sigma/a8592?lang=de&region=DE.
